# Imaging of brain electric field networks

**DOI:** 10.21203/rs.3.rs-2432269/v2

**Published:** 2024-04-12

**Authors:** Lawrence R. Frank, Vitaly L. Galinsky, Olave Krigolson, Susan F. Tapert, Stephan Bickel, Antigona Martinez

**Affiliations:** 1 Center for Scientific Computation in Imaging, UC San Diego, La Jolla, CA, USA; 2 7Center for Functional MRI, UC San Diego, La Jolla, CA, USA; 3 Centre for Biomedical Research, University of Victoria, Victoria, BC, Canada; 4 Dept of Psychiatry, UC San Diego, La Jolla, CA, USA; 5 Nathan Kline Institute, Orangeburg, NY, USA; 6 The Feinstein Institutes for Medical Research, Northwell Health, Manhasset, NY, USA

## Abstract

We present a method for direct imaging of the electric field networks in the human brain from electroencephalography (EEG) data with much higher temporal and spatial resolution than functional MRI (fMRI), without the concomitant distortions. The method is validated using simultaneous EEG/fMRI data in healthy subjects, intracranial EEG data in epilepsy patients, and in a direct comparison with standard EEG analysis in a well-established attention paradigm. The method is then demonstrated on a very large cohort of subjects performing a standard gambling task designed to activate the brain’s ‘reward circuit’. The technique uses the output from standard EEG systems and thus has potential for immediate benefit to a broad range of important basic scientific and clinical questions concerning brain electrical activity, but also provides an inexpensive and portable alternative to function MRI (fMRI).

## 1 Main

The human brain communicates internally through exceedingly complex spatial and temporal patterns of electrical signals. Although these signals can be measured using electrodes placed on the surface of the scalp (electroencephalography, or EEG), the ability to reconstruct the spatial and temporal patterns within the brain has been thwarted by the complexity of the inverse problem: What time- (or frequency-) dependent volumetric electrical signals throughout the brain are consistent with the signal measured on the two-dimensional surface of the scalp (1, 2)? There is a long-standing belief that it is not possible to detect and reconstruct electrical activity in sub-cortical regions deep within the brain from EEG due to inherent limitations of “volume conduction” (3). However, this is not actually a physical limitation, but rather a consequence of the incomplete nature of the standard model used to characterize the EEG signal. Despite the obviously highly dynamical nature of the electrical activity that occurs within the very inhomogeneous and anisotropic composition of brain tissue, current EEG data analysis methods are still based on the assumption that the average tissue bioelectric properties (e.g., the average permittivity ${\bar{\in }}_{0}$ and conductivity $\bar{\sigma }$) are sufficient to describe the electric fields *E* in the brain. This leads to the approximation $\left|{\bar{\in }}_{0}\partial E/\partial t|\ll |\bar{\sigma }E\right|$ (4) which in turn leads to the assumption that the time depedence *∂E/∂t* can be ignored in the “typical” frequency range of brain signals (5). This is the ubiquitous so-called “quasi-static” approximation (6).

In reality, it is precisely the anisotropic and inhomogeneous nature of brain tissue that must be taken into account in order to develop an accurate physical model of brain electromagnetic behavior, as we have described in our recently developed universal theory of brain waves called *weakly evanescent transverse cortical waves* (WETCOW) (7–9). The surprising consequence of this theory is the existence of electric field waves generated as a consequence of the complex tissue boundaries (e.g., surface waves) that permeate throughout the brain and are in precisely the frequency range of observed brain electrical activity. This theory explains the broad range of observed but seemingly disparate brain spatiotemporal electrical phenomena from extracellular spiking to cortical wave loops, all of which are predicated on the time dependence of the electric fields within the complex architecture of anisotropic and inhomeneous tissue within the brain. This theory is necessary to provide a solution to the EEG inverse problem which, as shown below, produces a reconstruction of brain electrical activity with high temporal resolution and spatial resolution that is comparable (or even exceeding that of) functional MRI.

## 2 Methods

The essence of our method (detailed in the Supplementary Material) is to reconstruct the electric field potential throughout the entire brain using our recently developed WETCOW wave model (7–9) constrained by MRI-defined tissue properties. This method is called *SPatially resolved EEG Constrained with Tissue properties by Regularized Entropy (SPECTRE)*. This approach solves a dynamic time-dependent construction of Maxwell’s wave equations of electromagnetism in an inhomogeneous and anisotropic medium and thus is distinctly different than standard so-called ‘source localization’ methods, as discussed in Section 6.3.

## 3 Validation

Validation of any neuroimaging methods is problematic because it is not possible to directly measure brain activity at every location in the brain. Nevertheless, three methods are obvious candidates for assessment of SPECTRE’s validity.

The first is comparison with functional MRI (fMRI), the current method of choice for whole brain spatial localization of brain activity. However, association of fMRI with a “standard” for EEG is problematic because it is not measuring electrical activity, but the magnetization changes in hemoglobin as blood becomes deoxygenated during brain activity. The timescale and location of these changes can be vastly different than those produced by EEG signals. Nevertheless, its capability of spatially localizing activated brain regions merits a comparison. The most direct comparison is between fMRI and EEG data collected simultaneously, which guarantees that the brain activity measured is identical in both experiments. Such “simultaneous fMRI/EEG” experiments are not particularly common as collecting EEG data within an MRI scanner during imaging is notoriously difficult, and the MR imaging procedure significantly distorts the EEG signal. However, a recent open-source study provides such data which is sufficient for our purposes.

A more direct method for validating the ability of SPECTRE to reconstruct localized electrical activity can be constructed from intra-cranial EEG (iEEG) recordings collected during epilepsy studies. Such measurements consist of specially designed EEG sensors distributed linearly along a probe that is inserted deep within a brain that has been exposed by surgical removal of a portion of the skull. By selecting only these electrodes near the brain surface from the full array of electrodes, we can synthesize an artificial surface distribution of electrodes to mimic a standard non-invasive EEG experiment (albeit with a limited coverage of the brain). We have access to such data through an ongoing study which enabled this method of validation as well.

Lastly, a comparison with current “source localization” methods would seem to be in order (10). This comparison turns out to be the most problematic as these methods all employ a very different, and quite limited, physical model for the EEG signal, and suffer from computational limitations as well. Despite attempts to make a reasonably valid comparison, it was determined that this was not possible, as described below.

### 3.1 Validation with simultaneous fMRI/EEG visual task

It is notoriously difficult to get high quality EEG data in simultaneous fMRI/EEG studies as the presence of the rapidly varying magnetic fields present in an fMRI acquisition distort the EEG signal. However, one recent open-source simultaneous fMRI/EEG study of a well controlled visual task (the periodic flashing checkerboard) on multiple subjects ((11), available from the Nathan Kline Institute) provides important data to address this question.

**Figure 1:**
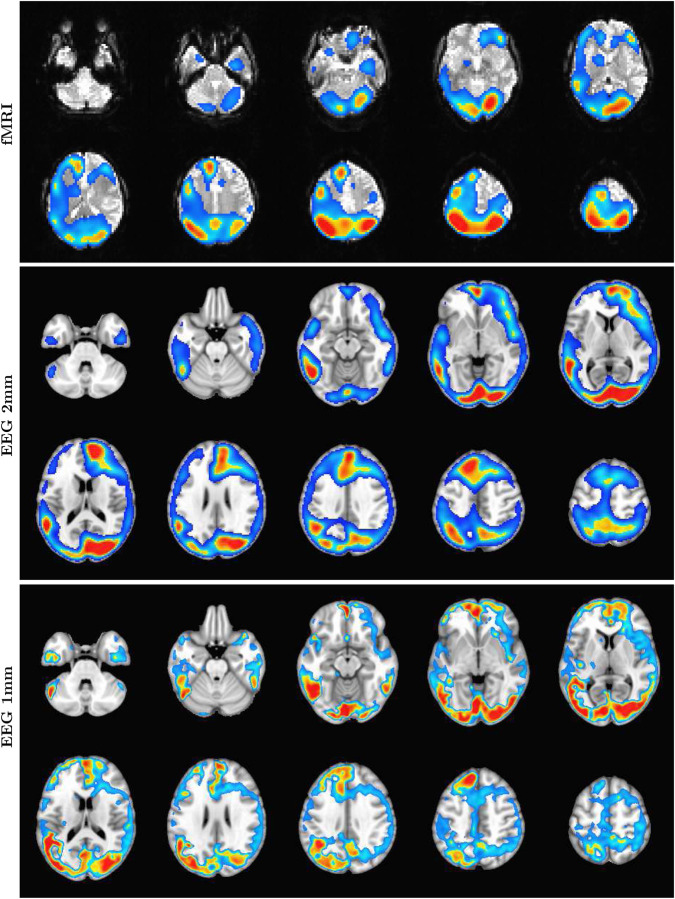
Comparison of EFD reconstructed fMRI activity (top) with SPECTRE reconstructed EEG at both 2mm (middle) and 1mm (bottom) spatial resolution (axial view) from a single representative subject from an open-source study with simultaneous fMRI and EEG (11). In both cases, the weighted sum of the power over all modes is shown. The task was a simple 8Hz flashing checkerboard with 4 on/off cycles.

The ability of SPECTRE to faithfully reconstruct the spatial distribution similar to fMRI is shown in Fig.1. Importantly, this comparison was performed on data from a single subject, since brain activity patterns can vary significantly between individual and averaging over multiple subjects obscures specific spatial variations important for validation. In the top rows of Fig. 1 is shown the fMRI EFD mode that automatically detects the activation in the primary visual cortex. In the middle row are shown the SPECTRE modes reconstructed using the 2mm MNI anatomical atlas, chosen because it was closest in resolution (2*mm*^3^) to the fMRI data (~ 3*mm*^3^). The very close correspondence between the spatial patterns is evident.

The bottom rows in Fig. 1 clearly demonstrate one of the most compelling, and perhaps surprising, aspects of SPECTRE - its ability to reconstruct activation at spatial resolution *significantly higher* than fMRI. This is a consequence of the SPECTRE reconstruction being based on the solution of the propagation of electromagnetic wave through specific tissue morphologies and bioelectric properties, provided by arbitrary resolution anatomical MRI data. The finer the resolution of the MRI scans, the more details can be available for the reconstruction. This is of course dependent upon the number and distribution of the EEG sensors, but certainly holds for the standard array configurations used in this paper.

**Figure 2:**
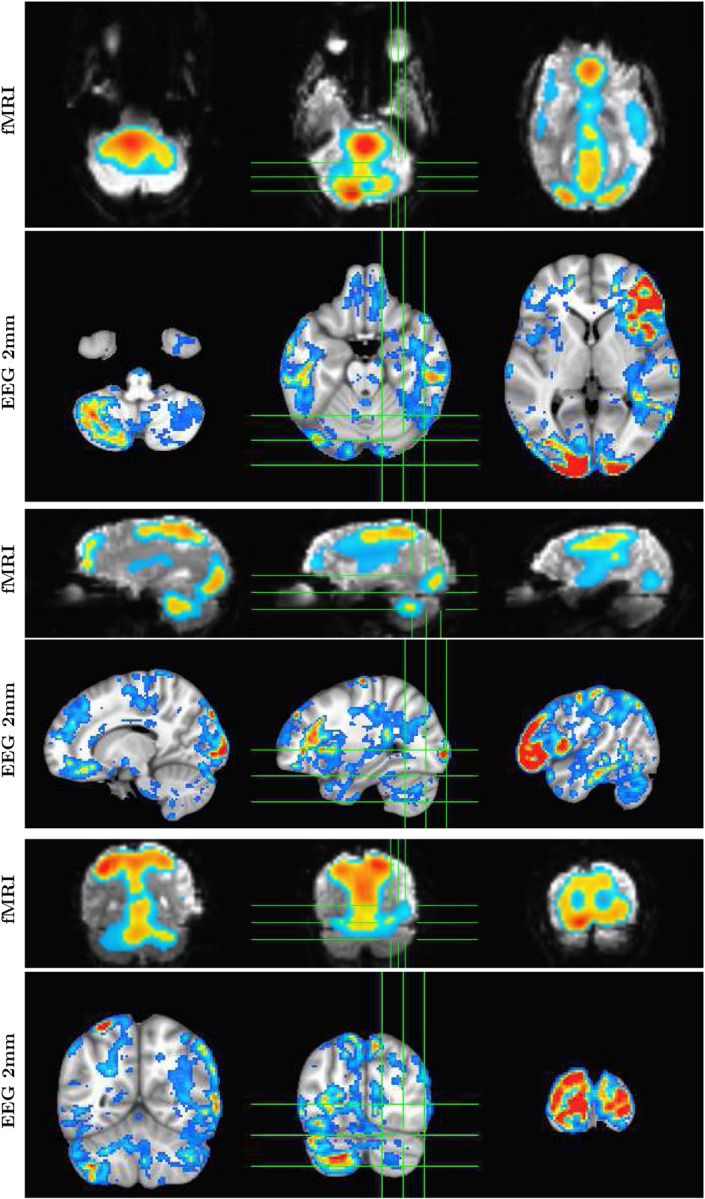
Comparison of EFD reconstructed fMRI activity (top) with SPECTRE reconstructed EEG at 2mm (bottom) from a single subject with simultaneous fMRI and EEG. (Same data source but different subject as in Fig. 1) demonstrating the fine spatial resolution produced by SPECTRE, and the ability to reconstruct activations in regions prone to severe distortions in fMRI, such as the frontal lobes.

Although it is an almost universally believed notion that EEG and fMRI are complementary because EEG has excellent temporal resolution but poor spatial resolution, while fMRI has poor temporal resolution but good spatial resolution, in fact SPECTRE EEG reconstructions can achieve much higher *intrinsic* temporal *and* spatial resolution. Moreover, because there are no spatial distortions in SPECTRE, this mitigates one of the aspects of fMRI that most confounds spatial localization through signal loss and non-linear geometric distortions. This is shown in Fig.2.

It should be noted that the ‘simple’ periodic flickering checkerboard stimulus not only activates the primary visual cortex but activates other visual and supplementary fields as well, as is evident from the activity patterns in Fig.1. A simple stimulus does not imply a simple activation pattern. This notion was a primary motivation for our development of the EFD method for fMRI (12). The activation mode reconstructions for both the fMRI and SPECTRE data are based on the EFD which detects complex non-linear interacting spatial-temporal modes of activity (12). Thus although the task is a ‘simple’ visual stimulation, our analysis is not expected to simply detect activity in only the visual cortex, as would be produced by a more standard regression approach (11), but in a more complex set of brain networks. Indeed, multiple EFD modes are produced, though we have only shown the one incorporating the primary visual cortex. As we have argued previously (12), EFD analysis is more sensitive than simple regression techniques to the complex brain activation patterns predicted by neuroscience, and less sensitive to erroneous identification of noise or non-independent modes than the independent component analysis (ICA) (12). Indeed, one of our observations from both the fMRI and EEG data used in this study (11) is the appearance of PFC activations associated with visual stimulation, which has been suggestive of conscious visual perception (13, 14). Addressing this question is beyond the scope of the current paper.

### 3.2 Validation with simultaneous fMRI/EEG attention paradigm

Simultaneous EEG/fMRI were collected from subjects within a standard clinical 3T MRI scanner (see Section 6.4.1 for details). The stimuli and paradigm are described in detail in (15). Briefly, bimodal stimuli consisting of short (~ 1*s*) streams of simple tones (600 and 1000 Hz) alternating at 10 Hz were delivered concurrently with phase-reversing (6Hz) checkerboard patterns presented at fixation. Participants were instructed to selectively attend to either the visual or auditory aspect of the bimodal stimulus and respond when the stream of stimuli in the attended modality ends.

**Figure 3:**
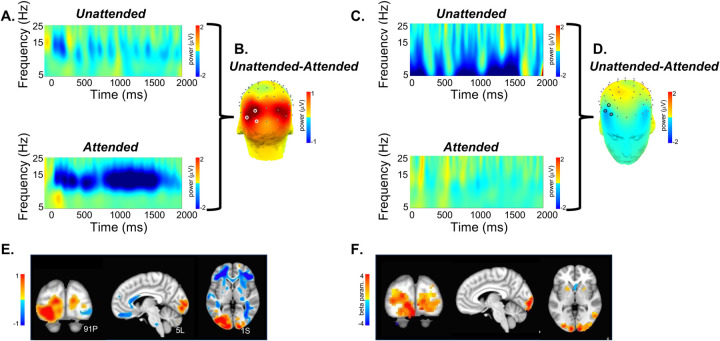
A. Baseline-corrected EEG activity from a single subject elicited by unattended (top) and attended (bottom) visual stimuli averaged across the cluster of 3 occipital electrode sites (PO7, PO3, O1) denoted in B. by white circles. Over the broad alpha frequency band (7–16Hz) there was a reduction in total power (from the pre- to post-stimulus latency interval) which was greater for attended, compared to unattended, visual stimuli. B. Scalp-topography of the mean difference in oscillatory (8–12Hz) activity for unattended minus attended visual stimuli across the 0–2000ms latency interval. As expected, attention modulated (reduced) the power of these oscillations over the visual cortex. C. As in A. for three frontal electrode sites (F6, F8, AF6) denoted in D. by black circles. In contrast to visual cortex, in bilateral frontal regions, unattended visual stimuli elicited a greater reduction of oscillatory activity between 5–10Hz (theta-alpha frequency). D. Frontal view of the unattended minus attended difference topography between 0–2000ms in the 8–12Hz frequency band. E. Source estimates derived from mean (baseline-corrected) oscillatory power between 0–2000ms and across 8–12Hz for the same subject shown in panels A-D, superimposed on the MNI template brain. Hot colors (yellow to red) indicate greater attention-related modulation (reduction) of activity and the inverse for warm colors (light to dark blue). F. BOLD signal (beta parameter estimate) contrasting activation to visual stimuli when attended versus activation to the same stimulus when unattended. Attention-related enhancement of the BOLD signal in visual cortex mirrors the reduction in alpha power obtained in the same subject using EEG.

**Figure 4:**
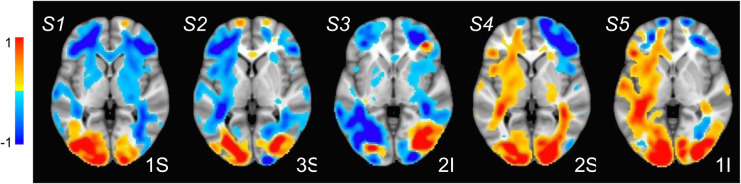
Estimated localization of neural sources for 8–12Hz oscillatory activity (unattended minus attended; 0–2000ms) for five participants (S1-S5). Colors are as in 1E. A prominent bilateral occipital source associated with increased attentional modulation is observable in all participants. A bilateral source localized in middle frontal cortex and indicating less modulation is also consistently observed across participants.

SPECTRE processing was performed in the alpha band. The appearance of visual stimuli elicited a reduction of ongoing alpha (7–14Hz) activity (“event-related desynchronization”, ERD) over occipital cortex, believed to occur when cortical regions are brought “on-line” for information processing (16). As in previous studies, e.g. (17), attended visual stimuli elicited increased (more negative) amplitude of the alpha ERD compared to unattended stimuli (Fig.3A,B). In contrast, unattended, compared to attended, visual stimuli elicited a greater reduction in ongoing spectral activity within the 5–15Hz frequency range over bilateral middle frontal cortex (Fig. 3C,D). We estimated the neural sources of these attention-related modulations of oscillatory activity across the 8–12Hz frequency band which encompassed both the occipital and frontal activities (Fig.3E). Their anatomical localization was remarkably consistent across several individuals (Fig.4).

**Figure 5:**
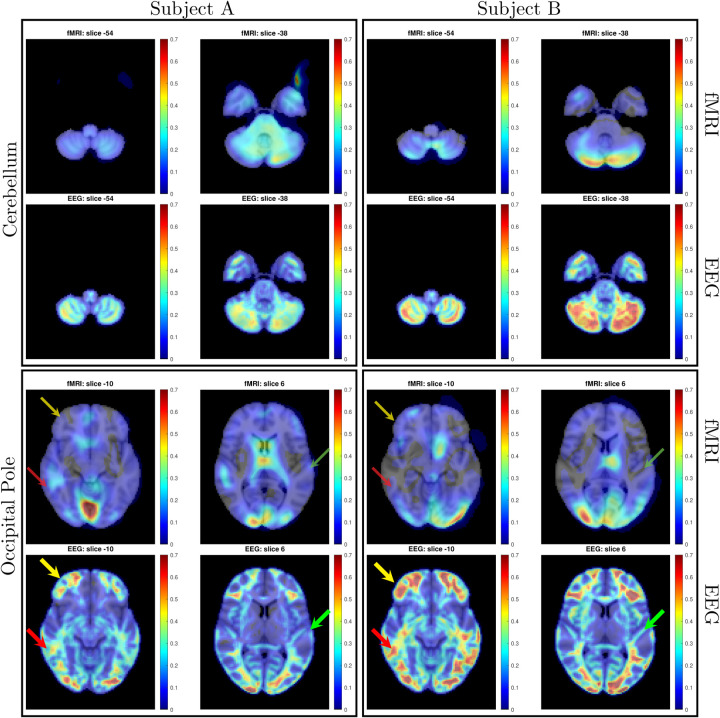
Direct comparison of activation maps from two participants (Subject A, left; Subject B, right) in the bimodal (auditory + visual) stimulation paradigm described for Figures 3 and 4. In each subject, two brain regions – the cerebellum and the occipital pole (top and bottom rows, respectively), were delineated based on the MNI atlas and EFD activation maps were correlated across these entire regions. Correlation coefficients were as follows: for Subject A, cerebellum=0.74, occipital pole=0.70; for Subject B, cerebellum=0.70, occipital pole=0.84. Correlations were computed only for regions exhibiting activation levels above 0.1. In contrast to fMRI, the SPECTRE technique identified robust activations in bilateral middle and inferior frontal cortex (indicated by yellow arrows) and middle temporal cortex (red arrows). It also discerned activations along the superior temporal cortex, including areas encompassing the primary auditory cortex (green arrows).

A direct comparison of the activation maps derived from both fMRI and EEG using SPECTRE for single study within two subjects (i.e. without any average over studies or subjects) is shown in Fig.5. The comparison is made by choosing specific regions of interest defined in the MNI atlas (occipital cortex and cerebellum) and correlating the activation maps derived from EFD for fMRI and SPECTRE from EEG. Comparison of the similarity of activated regions in individual subjects is generally a non-trivial problem. This is particularly true in the current case where the spatial distortions in fMRI (and lack of them in SPECTRE) make measures such as mean-squared error difficult to interpret. Therefore computation of the correlation coefficient over a predefined atlas ROI is a reasonable conservative meausure. The high correlation coefficients between the maps Fig.5 are therefore indicative of the consistency between the fMRI and SPECTRE results in the ROI. Note that this does *not* imply similarity over the entire region shown. Indeed, the SPECTRE results show enhanced sensivity to activation in regions not seen in the fMRI.

### 3.3 Validation through Intra-Cranial EEG recordings

**Figure 6:**
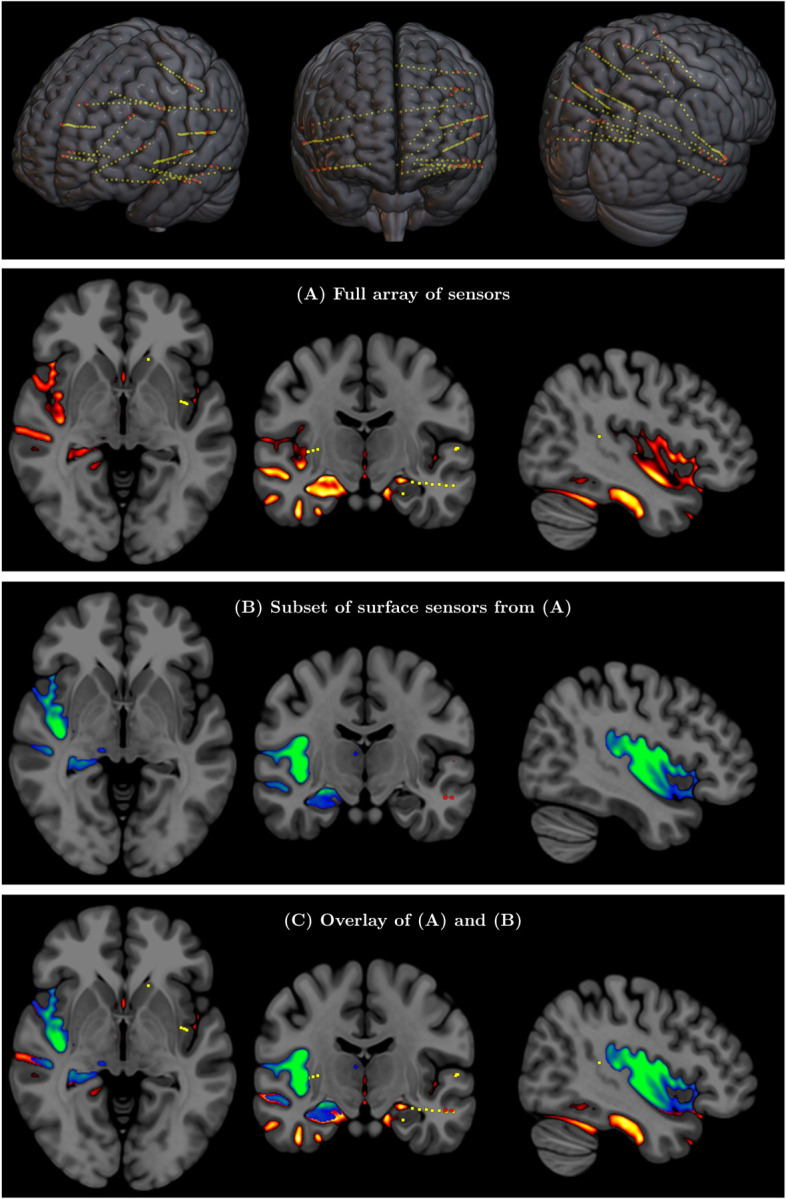
(Top row) Full array of intra-cranial EEG contacts from a recording in a medically refractory epilepsy patient (yellow dots). Red dots indicate subset of surface-only electrodes to mimic a standard non-invasive (i.e., extra-cranial) EEG study. SPECTRE *α* band reconstruction from (A) full array of intra-cranial EEG sensors from an epilepsy study (yellow dots) in top row and (B) from subset of surface electrodes (red dots) in top figure. (C) Overlay of (A) and (B) validating that the surface based is correctly reconstructing the local electric field potential detected by the intra-cranial electrodes.

While comparison with fMRI can validate the correct detection of activated brain regions and networks, as shown in the previous section, it cannot inform the question of correct detection of electrical signals, since fMRI is based on a completely different contrast mechanism related to blood oxygenation. A direct validation of SPECTRE’s ability to faithfully reconstruct deep electromagnetic activity is, to our knowledge, only achievable with one type of data: intracranial EEG (iEEG) recordings such as those used in medically refractory epilepsy patients for seizure onset localization where the electrodes are known to be adjacent to the site of electrical activity (18, 19). We analyzed an iEEG recording of a seizure localized in the left mesial temporal region acquired at Northwell Health, NY. All implanted electrodes are shown in Fig.6 (Top row) with each yellow dot depicting one recording contact. Comparing the SPECTRE reconstruction using all of the sensor data with one using only a subset of the data comprised of only the sensors on the surface of the brain (red dots in Fig.6 (Top row)) allows the quantitative assessment of how closely the results from a set of surface electrodes correspond to those produced by intra-cranial measurements recording signal very close to the sources. The results are shown for the alpha frequency band in Fig.6 and reveal a very close correspondance between the SPECTRE mode reconstruction.

## 4 Investigation of the ‘reward circuit’

Having validated the SPECTRE method directly with simultaneous fMRI/EEG, iEEG, and an attention paradigm, we investigated the ability of SPECTRE to faithfully reconstruct the well known neural ‘reward circuit’ that is one of the most important in understanding human cognition, emotion, and behavior (20–22) and is of great clinical significance in the understanding of addiction (23, 24), mood disorders (25, 26), and a variety of other conditions (27).

We demonstrate that SPECTRE using standard EEG data can accurately map human reward pathways akin to results previously only seen via fMRI. Indeed, fMRI results have highlighted a reward system within the brain that includes midbrain dopamine producing regions (the substansia nigra pars compacta, the ventral tegmental area), the ventral striatum, and multiple regions within the human prefrontal cortex (28). Other research using fMRI and source localization of EEG data suggests that the anterior cingulate cortex also plays a key role in reward processing (29). In a unifying theory, it has been proposed that all the aforementioned regions work together as a neural system for the optimization of reward driven behavioral change (i.e., reinforcement leaning; (30).)

This is of particular clinical significance because addictive behaviors have long been known to be subserved by specific brain regions operating in concert as the reward circuit (31–35). The reward circuit is involved in processing rewarding stimuli of any sort and in drug addiction, substances of abuse (e.g., amphetamine) increase dopamine release in a protracted and less regulated manner as compared to typical stimuli, resulting in synaptic plasticity, and altered functioning of this circuit over time.

For our analysis we used large gambling task dataset that includes 500 participants available for download from www.osf.io/65×4v/. The details of the dataset and an extensive analysis using standard EEG analysis methodologies are presented in (36). The relevant information from this study is presented in Supplementary Materials Section 6.4.2.

**Figure 7:**
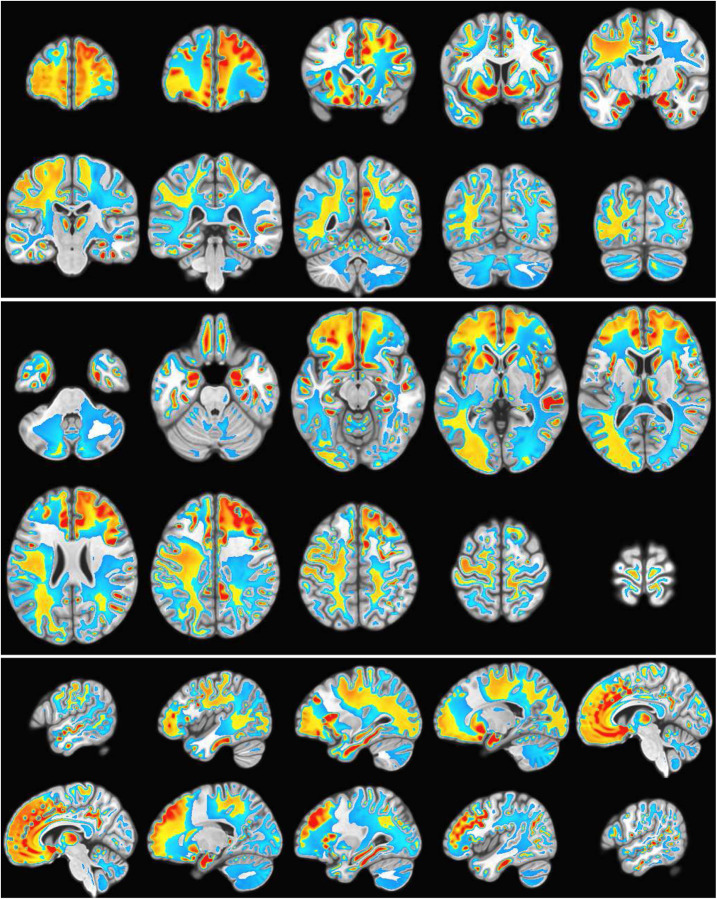
Gambling task EEG from 500 subject cohort. Alpha power of the weighted summed over the first *n* = 10 SPECTRE modes ${\tilde{\Psi }}_{10}$. Activation in key regions of the reward circuit, including the frontal lobes, paracingulate gyrus, accumbens, and amygdala are clearly evident. Negative activation (i.e., deactivation) is evident in the supplementary motor cortex and the left temporal-parietal regions.

For each subject trial *n* = 10 power modes were calculated and summed to form the single space-time SPECTRE mode ${\tilde{\Psi }}_{10}$ (see Section 6.1.2). Fig. 7 shows three orthogonal slices of the difference in EFD power summed over all modes between conditions, averaged over all subjects. Activation in key regions of the reward circuit, including the frontal lobes, anterior cingulate gyrus, accumbens, and amygdala are clearly evident. Strong negative activation (i.e., deactivation) is evident in several structures, including the supplementary motor cortex, and the parietal operculum cortex. Activation is also apparent in the lingual gyrus and around the calcarine fissure and, as expected, in bilateral subcortical structures.

**Figure 8:**
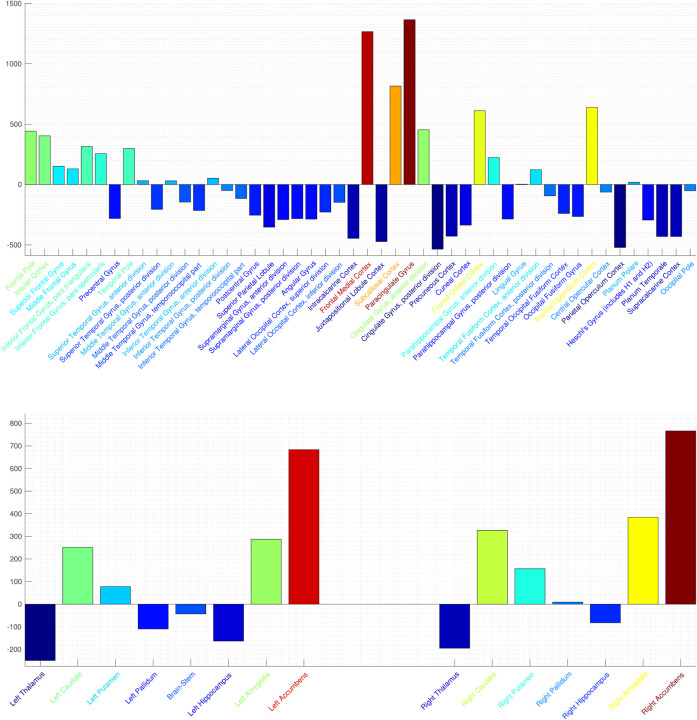
SPECTRE power per brain region in the Harvard-Oxford 2mm cortical (top) and subcortical (bottom) atlases. Colormap is from hot/yellow (activated) to blue (de-activated). Activation in key regions of the reward circuit, including the frontal lobes, paracingulate gyrus, subcallosal cortex/nucleus accumbens, and amygdala are clearly evident. Negative activation (i.e., deactivation) is evident in the supplementary motor area, posterior cingulate, and thalamus. Activation of the important reward element accumbens is evident in the bottom plot. Also of note is the relatively similar activation in the bilateral subcortical elements.

In Fig.8 is shown the power per brain regions as defined by the Harvard-Oxford 2mm cortical (top) and subcortical (bottom) atlases. In the cortical regions (top), strong activation is apparent in the frontal cortex (medial, orbital, operculum), cingulate gyrus, paracingulate gyrus, and unsular cortex. Activation in the accumbens is apparent from the data in the sub-cortical atlas Fig.8 (bottom). These activated regions are consistent with the known elements of the human brain reward circuitry.

**Figure 9:**
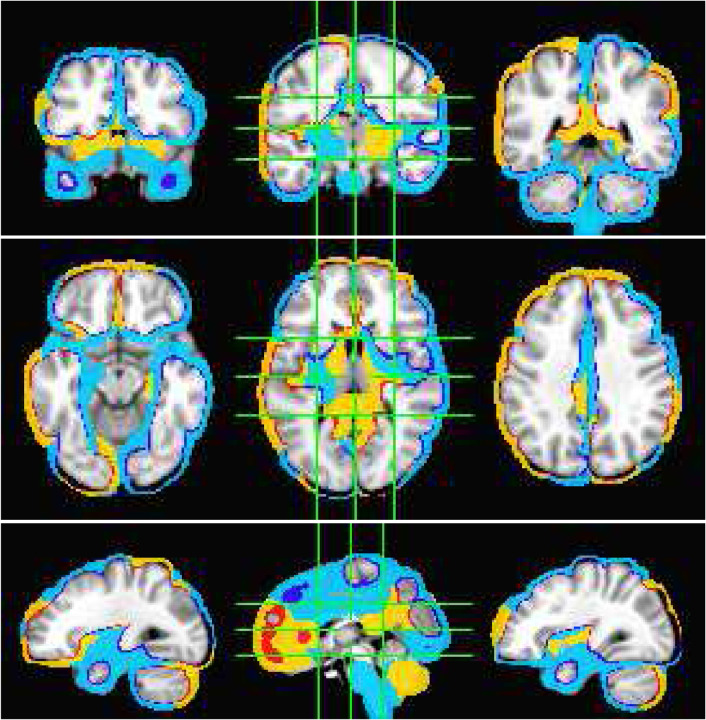
Statistical significance. t-statistic between the SPECTRE power modes pre- and poststimulus reward experiment. Calculations were performed using the standard AFNI 3dttest++ algorithm. Yellow/red color reflects positive changes, blue color reflects negative changes. Significance threshold was *p* = 10^−8^, indicating strong statistical significance.

Images of statistical significance (*p* < .0001) are shown in Fig.9. It should be noted that the determination of statistical significance with SPECTRE by ‘traditional’ methods is potentially misleading as they will tend to *underestimate* activation significance. The estimation of the modes in SPECTRE employs EFD (12, 37) which is a probabilistic formulation that *by construction* incorporates space-time neighborhood connectivity so that spatially and temporally coherent patterns (“clusters”) are more probable. Traditional methods have the option for “clustering” regions of activation *post-hoc* into their general class of techniques called “boot-strapping” or “permutation inference”. Cluster post-detection of an activation is incommensurate with our view of the estimation process wherein the clustering in space-time is a key component indicator of high-probability regions of space-time. Spatially and temporally coherent patterns maybe of low amplitude with apparent low significance by traditional means, but those intensities are within a mode that contains very high significance in cortical regions (e.g., Fig.9) which is predicted by the WETCOW model. Thus the entire SPECTRE mode, including the somewhat diffuse lower intensity regions, is significant.

## 5 Discussion and Conclusion

The ultimate goal of functional neuroimaging is to non-invasively detect and quantify the spatial and temporal variations in brain activity. Functional MRI (fMRI) and EEG have emerged as the most ubiquitous methods because they offer two important and complementary outcomes. fMRI can non-invasively detect complex spatial and relatively low frequency temporal patterns of activity related to local BOLD changes while EEG can directly detect electrical activity but without the capability of accurate spatial localization.

Ideally, one would measure the electrical activity of the brain at high temporal resolution, as done by EEG, but with the spatial localization capabilities of fMRI. However, there has been a long-standing belief that this is not possible, due to the ‘volume-conductance’ problem (3). In this paper we have shown that this is not an actual physical limitation, as it is always presented, but an artificial constraint stemming from model for how electromagnetic wave propagate within the brain that is overly simplified by neglecting local tissue anisotropy and inhomogeneity. Our recently developed WETCOW model incorporates these tissue characteristics into the electromagnetic field equations (i.e., Maxwell’s equations), which predicts the existence of previously undiscovered waves generated precisely as a consequence of the tissue anisotropy and inhomogeity. Unlike standard electromagnetic waves characterized by a frequencies *ω* that are proportional to their spatial frequency (or wavenumber) *k*, (i.e., *ω* ~ *k*) the WETCOW waves have an inverse relationship *ω* ~ 1*/k*. This results in waves that can permeate throughout the brain, not necessarily along neuronal pathways, that are in precisely the spatial and temporal (frequency) range of observed brain electrical activity. This model provides the physical model upon which the SPECTRE is based, using tissue models derived from standard high resolution anatomical MRI data to reconstruct the modes of brain electrical activity throughout the entire brain volume.

The SPECTRE reconstruction of EEG data provides obvious significant advantages over fMRI in temporal resolution, since EEG data has very high intrinsic temporal resolution (~ 1*ms*) necessary to capture rapidly varying electric field variations. Moreover, the SPECTRE algorithm can specify what frequency ranges to interrogate, providing a highly flexible analysis framework for focussed investigation of particular frequency bands of interest. On the contrary, even rapid fMRI acquisition is intrinsically limited by the temporal evolution of the contrast mechanism, the BOLD signal, which is related to blood flow and thus of quite low frequency (~ 1*Hz*).

While the advantages of SPECTRE over fMRI in temporal resolution are clear, what is perhaps surprising is its advantages in *spatial* resolution. The inverse solution that estimates the electric field potential from the EEG data is based on a physical model of wave propagation from tissues whose composition and geometry are derived from high resolution anatomical MRI data. The final resolution of the SPECTRE electric field modes is that of the anatomical data which is typically significantly higher (~ .5 – 1*mm*)) than the resolution of an fMRI image (~ 2*mm*). (There are, of course, limitations depending on the number of electrodes in the EEG system.)

But it is also important to recognize that the question of resolution in fMRI is not just a question of the prescribed image resolution of the acquisition. The BOLD physical mechanism that generates the fMRI contrast is a subtle variation in the magnetic susceptibility which causes variations in the local magnetic field, that in turn alters the local signal. fMRI acquisitions are specifically designed to accentuate this effect in order to make is observable. Unfortunately, local magnetic field variations unrelated to the BOLD mechanism, in particular strong magnetic susceptibility variations due to air/tissue boundaries such as those in the sinus cavities, cause severe non-linear image distortions that effectively alter the location and shape of the affected image volume elements (voxels). This makes even the definition of ‘resolution’ problematic, as it is essentially a spatially non-linearly varying function. Such effects are absent from EEG, which is simply a set of receiving electrodes (albeit not without its own source of artifacts) (3). The SPECTRE reconstruction uses high resolution MRI data acquired with techniques specifically designed to be insensitive to these magnetic susceptibility distortions and thus of very high spatial fidelity.

In this paper, we have successfully validated the SPECTRE method using simultaneous fMRI/EEG experiments. The results not only affirmed SPECTRE’s capability to accurately reconstruct spatial distributions of neural activity from EEG data, in alignment with the concurrently acquired fMRI data, but also revealed its efficacy in identifying robust activations across subjects that were not detectable with fMRI alone. These findings underscore SPECTRE’s potential to significantly enhance the sensitivity and scope of neuroimaging analyses. Further validation was performed using intra-cranial EEG measurements from an epilepsy study with reconstruction of data from a subset of sensors on the surface of the brain were shown to be consistent with the reconstruction from all the sensors, including those directly next to the activity source. The application of SPECTRE to high resolution EEG data during a gambling task demonstrated its ability to reconstruct a well-known and important brain circuit (20–22 ,24, 31, 32, 38) that has previously only been detected using fMRI. The analysis revealed significant differences in the brain networks in the alpha range 8 – 12Hz, consistent with previous spatially resolved fMRI experiments but the analysis is easily carried out in any user-defined frequency ranges of interest (39–42), which will be the subject of future work. The SPECTRE methodology is applicable to any EEG study and thus holds promise for a wide range of ongoing studies of basic neuroscience of reward mechanisms and in clinical applications such as addiction.

The implications for spatially resolved EEG are important not only from a scientific perspective, but from a practical perspective as well. fMRI is a much more involved and expensive procedure, requiring highly trained research or clinical applications specialists in specially designed facilities, and subjecting the subjects to a much more claustrophobic and restricted environment, with the safety concerns always present in MRI imaging experiments. On the contrary, the portability, safety, and relative ease of EEG experiments, which can be carried out in a standard research or clinical office, makes it very attractive. The high spatial and temporal resolution capabilities provided by SPECTRE to standard EEG data offer the possibility of more detailed investigations of brain activity in a wide range of both basic research and clinical settings. This method also has important implications for the democratization of medicine worldwide where there are many populations for which advanced technologies such as fMRI are prohibitive because of cost, citing issues for large specialized equipment, and lack of highly trained personnel.

### Human Subjects

All participants provided informed consent approved by the University of Victoria’s Human Research Ethics Board. The iEEG data was recorded in drug-resistant epilepsy patients under-going invasive EEG monitoring at the North Shore University Hospital (Manhasset, NY 11030, USA) for seizure onset localization. All patients provided informed written consent according to a protocol approved by the Institutional Review Board of the Feinstein Institutes for Medical Research in accordance with the Declaration of Helsinki.

## Supplementary Material

1

## Data Availability

The data that support the findings of this study are available from the corresponding author upon reasonable request.
