# Imaging of brain electric field networks with spatially resolved EEG

**DOI:** 10.21203/rs.3.rs-2432269/v3

**Published:** 2025-03-12

**Authors:** Lawrence R. Frank, Vitaly L. Galinsky, Olave Krigolson, Susan F. Tapert, Stephan Bickel, Antigona Martinez

**Affiliations:** a Center for Scientific Computation in Imaging, Department of Radiology, University of California San Diego, 8950 Villa La Jolla Dr., Suite B227, La Jolla, CA 92037, USA; b Center for Functional MRI, Department of Radiology, University of California San Diego, 9500 Gilman Dr., #0677, La Jolla, CA 92093-0677, USA; c Centre for Biomedical Research, University of Victoria, Victoria, BC, Canada; d Dept of Psychiatry, UC San Diego, La Jolla, CA 92093-0407, USA; e Nathan Kline Institute, Orangeburg, NY USA; f The Feinstein Institutes for Medical Research, Northwell Health, Manhasset, NY, USA

**Keywords:** Electroencephalography (EEG), Functional Magnetic Resonance Imaging (fMRI), Neuroimaging, Brain Waves, Entropy Field Decomposition (EFD), Spatially Resolved EEG Constrained with Tissue properties by Regularized Entropy (SPECTRE), brain connectivity

## Abstract

We present a method for spatially resolving the electric field potential throughout the entire volume of the human brain from electroencephalography (EEG) data. The method is *not* a variation of the well-known ’source reconstruction’ methods, but rather a direct solution to the EEG inverse problem based on our recently developed model for brain waves that demonstrates the inadequacy of the standard ’quasi-static approximation’ that has fostered the belief that such a reconstruction is not physically possible. The method retains the high temporal/frequency resolution of EEG yet has spatial resolution comparable to (or better than) functional MRI (fMRI), without its significant inherent limitations. The method is validated using simultaneous EEG/fMRI data in healthy subjects, intracranial EEG data in epilepsy patients, comparison with numerical simulations, and a direct comparison with standard state-of-the-art EEG analysis in a well-established attention paradigm. The method is then demonstrated on a very large cohort of subjects performing a standard gambling task designed to activate the brain’s ’reward circuit’. The technique uses the output from standard extant EEG systems and thus has potential for immediate benefit to a broad range of important basic scientific and clinical questions concerning brain electrical activity. By offering an inexpensive and portable alternative to fMRI, it provides a realistic methodology to efficiently promote the democratization of medicine.

## 1. Introduction

The human brain communicates internally through exceedingly complex spatial and temporal patterns of electrical signals. Although these signals can be measured using electrodes placed on the surface of the scalp (electroencephalography, or EEG), the ability to reconstruct the spatial and temporal patterns within the brain has been thwarted by the complexity of the inverse problem: What time- (or frequency-) dependent volumetric electrical signals throughout the brain are consistent with the signal measured on the two-dimensional surface of the scalp [1, 2]? There is a long-standing belief that it is not possible to detect and reconstruct electrical activity in sub-cortical regions deep within the brain from EEG due to inherent limitations of “volume conduction” [3]. However, this is not actually a physical limitation, but rather a consequence of the incomplete nature of the standard model used to characterize the EEG signal. Despite the obviously highly dynamical nature of the electrical activity that occurs within the very inhomogeneous and anisotropic composition of brain tissue, current EEG data analysis methods are still based on the assumption that the average tissue bioelectric properties (e.g., the average permittivity ${\overset{‾}{\epsilon }}_{0}$ and conductivity $\overset{‾}{\sigma }$) are sufficient to describe the electric fields E in the brain. This leads to the approximation $\left|{\overset{‾}{\epsilon }}_{0}\partial E/\partial t\right|\ll |\overset{‾}{\sigma }E|$ [4] which in turn leads to the assumption that the time depedence ∂E/∂t can be ignored in the "typical" frequency range of brain signals [5]. This is the ubiquitous so-called "quasi-static" approximation [6, 7].

In reality, it is precisely the anisotropic and inhomogeneous nature of brain tissue that must be taken into account in order to develop an accurate physical model of brain electromagnetic behavior, as we have described in our recently developed universal theory of brain waves called *weakly evanescent transverse cortical waves* (WETCOW) [8–10]. The surprising consequence of this theory is the existence of electric field waves generated as a consequence of the complex tissue boundaries (e.g., surface waves) that permeate throughout the brain and are in precisely the frequency range of observed brain electrical activity. This theory explains the broad range of observed but seemingly disparate brain spatiotemporal electrical phenomena from extracellular spiking to cortical wave loops, all of which are predicated on the time dependence of the electric fields within the complex architecture of anisotropic and inhomeneous tissue within the brain. This theory is necessary to provide a solution to the EEG inverse problem which, as shown below, produces a reconstruction of brain electrical activity with high temporal resolution and spatial resolution that is comparable (or even exceeding that of) functional MRI.

## 2. Results

### A new physical theory of brain waves

#### Background

The fact that the brain produces electrical signals, or brain waves, has been known for over 150 years, and the first recording in humans using electroencephalography (EEG) was made almost 100 years ago. The pioneering work of Cajal in the late 19th Century established the neuron doctrine that the nervous system is made up of discrete individual cells (neurons), which is one of the central tenets of modern neuroscience. Neurons are known to generate electrical signals as a result of their ability to maintain a voltage difference across their membranes that generates an electrochemical pulse known as an action potential that can travel rapidly along the axon. Consequently, the majority of approaches to characterizing brain dynamical behavior are based on the assumption that signal propagation along well-known anatomically defined pathways, such as major neural fiber bundles, tracts, or groups of axons, should be sufficient to deduce the dynamical characteristics of brain activity at different spatiotemporal scales. Characterizing brain networks is important for understanding many aspects of brain function, from neural processes underlying cognition to aberrant brain electrical activity, such as seen in epileptic seizures.

However this view cannot explain the entire picture of observed brain activity propagation. Recently spatiotemporally organized, circular wave-like patterns of electrophysiological activity (*traveling waves* were described at the macroscopic (scalp EEG, MEEG) and mesoscopic scale (invasive EEG), in animal models and humans, and during cognitive tasks and sleep [11–13]. These findings represent a formidable challenge for current network theories to explain such a remarkable synchronization across a multitude of different local networks.

In the following sections, we introduce the basic physical problem, present the rationale behind the ubiquitous “quasi-static approximations” and detail why it is a poor model for brain activity, and the outline our recently develop more general universal theory of brain waves, and how it ultimately leads to a solution of the inverse problem for EEG data.

#### Maxwell’s Equations in the Brain

The general equations governing the propagation of electromagnetic waves are called Maxwell’s Equations [14] and in an inhomogeneous and anisotropic medium take the form

(1a) ∇⋅D=ρ

(1b) ∇⋅H=0

(1c) $$\nabla \times E=-\frac{\partial H}{\partial t}$$

(1d) $$\nabla \times H=J_{t}$$

where D=εE is the *electric displacement field* where the (scalar) *permittivity* ε takes into account the polarization of the dielectric material in the electric field E, H is the magnetic field instensity, and the *total current density* $J_{t}$ is given by the sum of the free current density $J_{f}$ and bound current density $J_{b}$:

(2) $$J_{\mathrm{total}}=J_{\mathrm{free}}+J_{\mathrm{bound}}=J_{\mathrm{Conductive}}+J_{\mathrm{Displacement}}$$

where

(3) $$J_{C}=\sigma \cdot E\;,\;J_{D}=\frac{\partial D}{\partial t}$$

are the *conductive current* and *displacement current*, respectively.

This is the problem setup. To understand the electric fields in the brain, one needs to solve Maxwell's equations. At this point, the standard procedure (e.g., [3]) is the simply ignore the temporal variations in both the magnetic field and the electric field by setting $\frac{\partial H}{\partial t}=0$ and $\frac{\partial E}{\partial t}=0$. This simplified (1c) to ∇×E=0 and (1d) to $\nabla \times H=J_{C}$ since eliminating the time dependence of the electric field eliminates the displacment current: $J_{D}=\frac{\partial D}{\partial t}=\varepsilon \frac{\partial E}{\partial t}=0$. This is the so-called *quasi-static approximation* [6, 7] ubiquitious in EEG analysis methods.

What are the justifications for these simplifications? It turns that in biological tissues, the inductive effects are small are negligible [15] so that eliminating the time-dependence of the magnetic field is indeed justified. This is important as the simplified form of (1c) implies, for simply vector relations that the electric field can be written in terms of a *field potential* ϕ since

(4) ∇×E=0⇒E=-∇ϕ

Solving for the electric field E is then equivalent to solving for ϕ. And we can ignore any magnetic field effects.

However, the assumption that the electric field does not vary with time is *not* justified in biological materials [15] and therefore the expression (1d) is correct as is - the displacement current must be retained. Maxwell’s equations in the brain thus take on a somewhat odd configuration in that they are, in the standard physics parlance, *magnetostatic* but not *electrostatic*.

It is somewhat ironic that the introduction of the displacement current, which was in some sense Maxwell’s greatest insight, and the final piece of the puzzle in solve the equations of electromagnetism, turns out to be the key to the puzzle of brain activity, where it had once again been ignored.

#### Consequences of the Quasi-Static Approximation

Because of the ubiquity of the quasi-static approximation, it is worth pausing here to consider its consequences, since in our view they have led to confusion in the understanding brain electrical activity and the problem of EEG reconstruction.

$$\left.\begin{array}{r} E=-\nabla \phi \\ \nabla \cdot E=\rho /\varepsilon \end{array}\right\}\Rightarrow \nabla^{2}\phi =\frac{\rho }{\varepsilon }$$

which is Poisson's equation and relates the electric field potential ϕ to 'sources' ρ. The most striking aspect of this solution, though it was the obvious endpoint by construction, is that there is no time-depence in the solution. One would have guessed this to be a giant red flag for the description of brain electrical activity but the persistence of this approach has nevertheless be tenacious. Consequently, from the perspective of EEG reconstruction, the problem is framed in terms of ’source reconstruction’. We note that these equations are also called the “quasi-static volume conduction” equations and therefore this problem is often referred to as the “volume conduction” problem.

There is also another massive source of confusion that is often used to justify the quasi-static approximation. The logic goes something like this. We know that the brain has, for example, alpha waves which for the sake of simplicity we will assume the frequency to be a typical value of ω=10Hz. For electromagnetic waves in a medium of permittivity ε, the wavelength of these wave is related to the velocity v of the waves as

(5) $$\lambda =\frac{v}{\omega }\;\mathrm{where}\;v=\frac{c}{\sqrt{\varepsilon }}$$

in which $c=3\times 10^{8}\;m/s$ is the speed of light. For a typical tissue permittivity ε=100 the wave velocity is $v=3\times 10^{7}\;m/s$ so that the wavelength is

(6) $$\lambda =\frac{v}{\omega }=\frac{3\;\times \;10^{7}}{10}=3\times 10^{6}m=3000\;km$$

Because the wavelength is so much greater than the spatial dimensions of the head, there can be no appreciable phase difference anywhere in the head and electromagnetic wave propagation effects can be ignored. Indeed, this is true - there are effectively no EM wave propagation effects in the brain. But that argument is *not* a justification for the assumption of a time-independent electric field. Indeed, the logic is backwards. One must first solve Maxwell’s equations under the proper conditions, then eliminate contributions that appear insignificant.

Indeed, if one simply assumes the absence of free charges, Maxwell's equation (1c) and (1d) in a medium of permittivity ε and permeability μ combine to give

(7) $$\nabla^{2}E-\frac{1}{v^{2}}\frac{\partial^{2}E}{\partial^{2}t}=0\;\Rightarrow \;E(r,t)=E_{0}e^{i(k\cdot r-\omega t)}$$

the solution to which are complex plane waves, which in turn implies the *dispersion relation* ω=v|k|. As we will show below, the dispersion relation derived from the correct version of Maxwell’s equations is quite different and provides a key insight into the interesting characteristics of brain waves.

The fact that observed alpha wave have velocities many orders of magnitude slower than EM alpha waves should be an obvious clue that something else is going on. This much is recognized in that they are ascribed to ill-defined concepts such as “neuronal oscillations”. But a consequence of that should be a reexamination of the Maxwell’s equation in light of these experimental observation. We will do that in the next section and demonstrate that these ’slow’ wave are not mysterious at all, but a direct consequence of the displacement current and the inhomogeity and anisotropic of the tissues. They are not EM waves, but surface waves.

#### The General Solution: WETCOW theory

In this section we provide a brief outline the more detailed theoretical description in [8, 9]. From the above discussion, the proper form of Maxwell’s equation to solve, from (1) and (3), is

$$\left.\begin{array}{ll} \nabla \cdot D & =\rho \\ \nabla \times H & =\sigma \cdot J_{c}+\frac{\partial D}{\partial t} \end{array}\right\}\Rightarrow \frac{\partial \rho }{\partial t}+\nabla \cdot J_{c}$$

9where the RHS is a statement of *charge continuity*. These equation along with (4) and (3) give the charge continuity equation in terms of the quantity of interest, the electric field potential ϕ:

(8) $$\frac{\partial }{\partial t}\left(\nabla^{2}\phi \right)=-\nabla \cdot \Sigma \cdot \nabla \phi$$

where $\Sigma =\left\{\sigma_{ij}/\varepsilon \right\}$ is the scaled conductivity tensor. Thus the inclusion of the displacement current has produced a wave equation.

A simple linear wave analysis, i.e. substitution of ϕ∼exp[-i(k⋅r-Ωt)], where k is the wavenumber, r is the coordinate, Ω is the frequency and t is the time, gives the following complex dispersion relation, now written in tensor form where i,j={x,y,z} and repeated indices are summed:

(9) $$D(\Omega ,k)=-i\Omega k_{i}^{2}-\Sigma_{ij}k_{i}k_{j}-i\partial_{i}\Sigma_{ij}k_{j}=0,$$

which is composed of the real and imaginary components:

(10) $$\gamma \equiv I[\Omega ]=\Sigma_{ij}\frac{k_{i}k_{j}}{k^{2}}\;\omega \equiv R[\Omega ]=-\frac{\partial_{i}\Sigma_{ij}k_{j}}{k^{2}}$$

Several interesting features of this relation are worth noting. Because it is complex, it will result in both an oscillatory component (proportional to the frequency ω) and a decaying component (proportional to the decay rate γ). Both γ and ω are functions of the tissue parameters through $\Sigma_{ij}$ so there is a direct connection between tissue properties and the wave dynamics. The tissue properties are encoded in the tensor $\Sigma_{ij}$ so spatial variations due either to inhomogeneity or anisotropy will also influence wave propagation. But perhaps the most interesting feature of this dispersion relation is that ω∼1/k, and so quite different than the dispersion relation for EM waves. This has significant consequences for the nature of brain electrodynamics, as shown below.

These results bring us to a central important point. For typical low frequency (≲10Hz) *average* values of white and gray matter conductivity and permittivity (i.e. from [16, 17]) the decay rates (**??**) give strong wave damping, and no waves would be observed. For example, typical values for gray matter (GM) and white matter (WM) are $\varepsilon_{GM}=4.07\cdot 10^{7}\varepsilon_{0}$, $\varepsilon_{WM}=2.76\cdot 10^{7}\varepsilon_{0}$, $\sigma_{GM}=2.75\cdot 10^{-2}\;S/m$, $\sigma_{WM}=2.77\cdot 10^{-2}\;S/m$, where $\varepsilon_{0}=8.854187817\cdot 10^{-12}\;F/m$ is the vacuum permittivity) so the damping rate γ is in the range of 75–115 s^−1^ which would give strong wave damping. This leads immediately to the question of the effects of the anisotropy, which is encoded in the scaled conductivity tensor Σ. A full discussion of the effects of anisotropy is provided in [8, 9] but here we review the key novel and important finding of the general theory: the existence of previously unrecognized (at least theoretically) wave *transverse* to the fiber direction.

To see this, we take a very simple idealized tissue model: fibers are packed in a half space aligned in z direction and their number decreases in x direction in a relatively thin layer at the boundary. We assume that small cross fiber currents can be characterized by a small parameter ϵ and represent the conductivity tensor as

(11) $$\Sigma =\left(\begin{array}{ccc} \epsilon v & \epsilon v & \epsilon v\\ \epsilon v & \epsilon v & 0\\ \epsilon v & 0 & v \end{array}\right).$$

where v≡v(x). For the v(x) dependence we will assume that the conductivity is changing only through a relatively narrow layer at the boundary and the conductivity gradient is directed along x axis.

(12a) $$\left(\left[\partial_{t}+a\right]\partial_{z}^{2}+b\partial_{z}\right)\phi_{\|}=0\;,\;∼\epsilon^{0}\;(damped\;oscillator)$$

(12b) $$\left(\partial_{t}\partial_{y}^{2}+b\partial_{y}\right)\phi_{⊥}=0\;,\;∼\epsilon^{1}\;(wave\;equation)$$

where $a={\left.v(x)\right|}_{x_{0}}$ and $b={\left.\partial_{x}v(x)\right|}_{x_{0}}$ are considered constant evaluated at the boundary $x_{0}$, and where $\epsilon^{0}$ and $\epsilon^{1}$ denote the zeroth and the first orders of ϵ power. We emphasize that this approximation for a and b is specifically allowed because we are considering a thin boundary layer problem.

The first equation (12a) describes a potential along the fiber direction and is a damped oscillator equation that has a decaying solution. But the second equation (12b) describes a potential perpendicular to the fiber direction and does not include a damping term, hence it describes a pure wave-like solution that propagates in the thin layer transverse to the main fiber direction. Thus although this wave-like solution $\phi_{⊥}$ has a smaller amplitude than along the fiber action potential $\phi_{\|}$, it can nevertheless have a much longer lifetime. Such waves are called *weakly evanescent cortical waves*, or **WETCOW** for short.

Though these produce many interest effects, two aspects are most important for the current application. First, the decay rates γ for brain tissue is sufficiently small that waves can persist for time significantly longer than ’spiking’. The persistence of the stable waves can be characterized by the ratio of the decay rate to the frequency, which from simple geometric considerations from (10) for the longest waves (with the smallest amount of damping) with

$$\frac{\gamma }{\omega }=\frac{\Sigma_{ij}k_{i}k_{j}}{\partial_{i}\Sigma_{ij}k_{j}}\approx ∼0.02-0.04.$$

Anisotropy $\left(\Sigma_{⊥}<\Sigma_{\|}\right)$ will reduce this estimate even further (see [8, 9] for more details). In other words, anisotropy can result in decay rates that can vary from these maximum (homogeneous) values above, all the way down to 0, based on the direction of propagation, increasingly supporting the existence of transverse waves. Without taking anisotropy into account, that is, assuming the mean tissue values above as is done in the “standard model”, the decay is so rapid that transverse weakly evanescent waves are not supported.

Second, the inverse relationship between the frequency and wavelength means in the dispersion relation ((9)) means that waves can extend throughout the entire volume of the brain. One can also recognized immediately from (10) the existence of significant phase variations across the brain (characterized by both phase and group velocity) proportional to tensor products ∇⋅Σ and ∇⋅Σ⋅k) that characterize wave propagation normal to the conductivity gradient and thus normal to the fiber orientation. This contradicts the longstanding belief that there are no significant phase variations across the head. There are, but the are not due to EM waves, but WETCOW waves.

The existence of these waves has profound implications for the understanding of brain electrical activity and communications, and have been shown to explain a wide range of observed collective brain behaviors, including spiking in the extracellular space [8, 9], rapid signal synchronization [10] that provides a mechanism for learning and memory [18], and neuronal avalanches [19, 20]. And of course they require rethinking what is mean by a brain “network”, since signal propagation must now be considered not only along fiber pathways, but between structure that may not even be neuronally direction connected. But for the present purposes, they imply the existence of waves of electrical activity throughout the brain.

### Solution to the inverse EEG problem

#### Theory

The WETCOW theory predicts the exists of waves satisfying Maxwell’s equations in the brain where the morphology and tissue characteristics have been properly taken into account. The EEG inverse problem therefore involves estimating the electric field potential ϕ that satisfies Maxwell’s equations constructed with from the tissue properties of a particular brain, satisfying boundary conditions determined by the morphology of the brain, and consistent with measurements made in an array of electrodes on the surface of the brain. Our method for solving the inverse EEG problem can be summarized as follows. Given a standard EEG dataset from N electrodes and a high-resolution anatomical (HRA) MRI dataset with high contrast between gray matter (GM) and white matter (WM), the solution to the inverse EEG problem can be formulated as an approximation for the volumetric distribution of electrostatic potential inside the complex inhomogeneous and anisotropic tissues and complicated morphology of the MRI domain [21].

The solution to the EEG inverse problem entails solving (8) for the electric field potential ϕ. Taking the temporal Fourier transform (i.e. replacing ∂/∂t→-Iω, $I^{2}=-1$ where ω is the frequency), the electrostatic potential satisfies the equation in the Fourier (i.e., frequency) domain, and using the notation $\partial_{i}=\partial /\partial q_{i}$, $q_{i}=\{x,y,z\}$, can be written in tensor form as

(13) $$\left(\Sigma^{ij}-I\omega \varepsilon \delta^{ij}\right)\partial_{i}\partial_{j}\phi_{\omega }=\left[I\omega \left(\partial_{i}\varepsilon \right)\delta^{ij}-\left(\partial_{i}\Sigma^{ij}\right)\right]\partial_{j}\phi_{\omega }+{ℱ}_{\omega },$$

where δ is the Dirac delta function, and a summation is assumed over repeated indices. This can be expressed in the form $\overset{ˆ}{L}\phi_{\omega }=\overset{ˆ}{R}\phi_{\omega }+{\overset{ˆ}{ℱ}}_{\omega }$ in terms of the operators $\overset{ˆ}{L}\equiv \partial^{i}\partial_{i}$, a frequency dependent source term ${\overset{ˆ}{ℱ}}_{\omega }$ and the operator

$$\overset{ˆ}{R}\equiv \frac{\sigma \;+\;I\omega \varepsilon }{\sigma^{2}\;+\;\omega^{2}\varepsilon^{2}}\left[I\omega \left(\partial_{i}\varepsilon \right)\delta^{ij}-\left(\partial_{i}\Sigma^{ij}\right)-\left(\Sigma^{ij}-\sigma \delta^{ij}\right)\partial_{i}\right]\partial_{j}$$

where $\Sigma =\left\{\Sigma^{ij}\right\}$ is a local tissue conductivity tensor, $\sigma =Tr\Sigma /3=\Sigma_{i}^{i}/3$ is an isotropic local conductivity. Terms in square brackets show that the parts of $\overset{ˆ}{R}\phi_{\omega }$ can be interpreted in terms of different tissue characteristics and may be important for understanding the origin of sources of the electro-/magnetostatic signal detected by the EEG sensors. The first term $\left(\omega \left(\partial^{i}\varepsilon \right)\left(\partial_{i}\phi_{\omega }\right)\right)$ corresponds to areas with sudden change in permittivity, e.g. the WM/GM interface. The second term $\left(\left(\partial_{i}\Sigma^{ij}\right)\left(\partial_{j}\phi_{\omega }\right)\right)$ corresponds to regions where the conductivity gradient is the strongest, i.e. the GM/CSF (cerebral spinal fluid) boundary. Finally, the last term ($\left.\sum^{ij}\;\partial_{i}\partial_{j}\phi_{\omega }-\sigma \partial^{i}\partial_{i}\phi_{\omega }\right)$ includes areas with the strongest conductivity anisotropies, e.g. input from major WM tracts. The frequency and position dependent internal sources ${\overset{ˆ}{ℱ}}_{\omega }$ can be used to incorporate various nonlinear processes including multiple frequency effects of the efficient synchronization/desynchronization by brain waves or effects of their critical dynamics. This term is ignored in the current paper because they are higher order terms that complicate the processing (and intepretation) but do not substatially change the main results. They will be considered in future work.

#### Numerical Implementation

The inverse problem can be solved by constructing an approximate solution for the potential ϕ across an entire brain volume iteratively as $\overset{ˆ}{L}\phi_{\omega }^{\left(k\right)}=\overset{ˆ}{R}\phi_{\omega }^{\left(k-1\right)}$ and ${\tilde{\phi }}_{\omega }^{\left(K\right)}=\alpha_{K}\sum_{k=0}^{K}\phi_{\omega }^{\left(k\right)}$ [21]: where a single iteration forward solution is found using a Fourier-space pseudo-spectral approach [22]. The volumetric frequency-dependent potential ${\tilde{\phi }}_{\omega }^{\left(K\right)}$ is the central quantity of interest and it can be calculated over arbitrary frequency ranges $\omega =\omega_{1}\ldots \omega_{2}$, such as the standard frequency bands of interest in EEG. These potentials can then be converted to the time domain $\tilde{\phi }(t,x)$ and used for EFD analysis [23, 24]. Alternatively, as in this work, the estimated potentials $\tilde{\phi }(t,x)$ can be used in the joint estimation scheme presented in [25] as an additional modality $Q_{ij}^{E}$ in the intermodality coupling matrix ${𝒬}_{ij}$. (see Appendix Appendix A.6). The potential depends upon the electrical properties of the tissue permittivity, permeability, and conductivity. These parameters can be estimated from the HRA MRI data. Using JESTER, data from MRI can be used to define the complex brain tissue morphology and constrain the tissue specific values of Σ and ε. This procedure of inverting the WETCOW brain wave model constrained by MRI-defined tissue properties is called *SPatially resolved EEG Constrained with Tissue properties by Regularized Entropy (SPECTRE).*

An approximate pseudo-spectral solution for the potential ϕ was constructed across an entire brain volume using either MNI 2mm resolution (91×109×91 voxel dimensions), 1mm MNI resolution (182×218×182 voxel dimensions), or 0.7mm resolution (207×256×215 voxel dimentions). For the current study we only used the anatomical data for estimatition and assignment of different tissue types and no diffusion MRI data was used. To register between different modalities, including MNI, HRA, function MRI, etc., and to transform the tissue assignment into an appropriate space we used the SYMREG registration method [25].

The pseudo-spectral computational approach used in SPECTRE has some important advantages over the finite/boundary element approaches typically used for electrostatic modeling of brain activity [26–32]. It does not use surface meshes and so does not require limiting the location of activity sites to a small number of surfaces with fixed number of static dipole sources constrained to the surfaces. And the distribution of both electrostatic and geometric properties of the media (conductivity, permittivity, anisotropy, inhomogeneity - derived from the MRI data) are incorporated at every location throughout the volume. It is thus able to find a time dependent spatial distribution of the electrostatic potential at every space-time location of a multidimensional volume as a superposition of source inputs from every voxel of the same volume [21]. These traits allow it to model wave-like signal propagation inside the volume and can detect and characterize significantly more complex dynamical behavior of the sources of the electrostatic activity recorded at the sensor locations than traditional methods.

## Methods

### Summary of SPECTRE

The SPECTRE procedure can be summarized as follows. The data are the raw output from a standard EEG system and a high resolution anatomical (**HRA**) MRI image. A standard template (e.g., T1-weighted anatomical MRI Montreal Neurological Institute (MNI) [33]) is typically used so that an MRI acquisition is not required. The EEG data is registered to the HRA template using our non-linear *symplectomorphic registration* (**SYMREG**) [25]. The different tissue types and their geometry are determined from the HRA using our *spherical wave decomposition* (**SWD**) algorithm [34]. The estimated geometry is used to define the sampling points for the pseudo-spectral algorithm. The spatially variations in the tissue bioelectric properties are estimated from the spatial variation in the segmented tissue types. The pseudo-spectral algorithm is then solved for the electric field potential that best fits the raw EEG data at each electrode, constrained by the local tissue properties within the brain volume. The resulting potential field ϕ(x,t) is then decomposed into spatial-temporal modes using the EFD algorithm [23, 24] constrained by the anatomical atlas using JESTER [35].

In the present study, the HRA data were used to identify and segments the gray and white matter regions in order to define their separate geometries and the spatial variations in the tissue bioelectric properties (e.g., conductivity and permittivity) to go into the estimation of the field potential. However, SPECTRE is quite flexible in its ability to incorporate additional tissue information from other modalities, so as improved estimates of the local tissue conductivity tensor from diffusion MRI data (dMRI), where it available. We did not do so in the current study as the goal was to demonstrate the SPECTRE can be achieved without the necessity of acquiring any MRI data, which has significant practical implications.

The conductivity tensor is not exactly the same as the diffusion tensor in brain tissues, but they are closely related. While both tensors describe transport properties in brain tissue, they represent different physical processes. The conductivity tensor is often assumed to share the same eigenvectors as the diffusion tensor. There is a strong linear relationship between the conductivity and diffusion tensor eigenvalues, as supported by theoretical models and experimental measurements. For the current study we only used the anatomical data for estimatition and assignment of different tissue types and no diffusion MRI data was used.

To understand intuitively why SPECTRE is capable of reconstructing EM activity through the entire brain, including deep within subcortical structures, a simple idealized example is helpful. Consider two point current sources of different frequencies, one in the cortical layer close to the scalp, the second deep within the subcortical structures of the brain. Consider a single sensor placed on the scalp collinear with the two sources. Standard source localization methods will not see the deep source, since there is no frequency dependence, and the signal falloff is simply a function of the distance from the sensor. Therefore, the close source completely dominates the signal model. Since all tomographic imaging methods (e.g., MRI, CT, etc.) depend strongly on both the spatial and temporal sampling of the measured physical system, this effective invisibility of currents in the standard quasi-static model essentially precludes the solution of the true inverse EEG problem and necessitates the artificial construction of assumed dipole distribution on pre-chosen artificial internal structures. In contrast, in SPECTRE the sources are not dipoles, but frequency sources that extend throughout the entire brain volume subject to the boundary conditions imposed by both the tissues geometry and its spatially and frequency dependent properties. The surface electrodes are assumed to be sensing EM waves emanating from the entire brain across a broad frequency spectrum limited only by the sensors. Used in conjunction with an HRA MRI data that provides the spatial distribution of the frequency-dependent tissue electrical properties that constrain the possible solution, SPECTRE can invert the wave equations to provide an estimate of the spatiotemporal distribution of the electric field potential.

### Mode reconstruction

After estimating the non-linear spatially and temporally varying electric field potential, we still face the challenge of interpreting it, much like raw fMRI data must be analyzed to identify activation patterns. At this stage, the issues of fMRI and SPECTRE analysis are essentially the same. In general, this is a difficult task because brain activity exhibits a highly complex spatiotemporal structure. Conceptually, one can view “activation patterns” (or modes) as groups of spatially contiguous voxels sharing similar time courses, which may synchronize with other local regions located anywhere else in the brain. For example, the “default mode” network comprises several such contiguous regions—such as the dorsal medial prefrontal cortex, posterior cingulate cortex, precuneus, and angular gyrus—that operate together.

It is important to highlight several complicating factors inherent in time-dependent volumetric data from modern imaging systems, including neuroimaging scanners and meteorological radar. First, estimating spatiotemporal patterns requires addressing both spatial and temporal variations simultaneously. For example, it is not sufficient to analyze temporal patterns first and then spatial patterns after - a common practice in fMRI data analysis. One should not compute the correlation of a voxel with all other voxels (temporal analysis) and then use a clustering method (spatial analysis) to define a region of “significant” activity. Instead, the data should be viewed as space-time points whose space-time trajectories must be estimated as a whole. Second, time courses are typically neither simple nor periodic; they can follow virtually any form dictated by the underlying physical processes. Finally, data are often multiparametric, with parameters influencing (i.e., coupled to) one another. For instance, in fMRI blood flow and electrophysiology are coupled and influence each other.

The problem then becomes one of detecting the multiple modes in complex non-linear systems. We have addressed this problem previously in our development of the *entropy field decomposition* (EFD) method, which is a probabilistic framework for estimating spatial-temporal modes of complex non-linear systems containing multivariate interacting fields [23, 24, 36, 37]. These concepts are described in greater detail in Appendix Appendix A. It is formally based on a field-theoretic mathematical formulation of Bayes’ Theorem that enables the hierarchy of multiple orders of field interactions including coupling between fields. Its practical utility is enabled by incorporation of the theory of *entropy spectrum pathways* (ESP) [38], which uses the space-time correlations in each individual dataset to automatically select the very limited number of highly relevant field interactions. In short, it selects the configurations with maximum path entropy, summarized in the equilibrium (i.e., long time) distribution $\mu^{*}$. While each of these modes provides unique information on coherent spatio-temporal activity, for characterizing the total brain activity it is often most useful and efficient to sum these modes.

A strength of the EFD method is that it uses prior information contained in individual datasets - there are no training datasets or averages across datasets - just the prior information contained within the single dataset of interest. This method has shown utility in resting state fMRI data [24] and in meteorology in the application to severe local storms, in particular tornadic supercells [36]. The fact that this method uses prior information embedded within single datasets without the need for any ’training’ is of significance to clinical studies in which important individual variations can be lost in the averaging process. It is also particularly important in the current paper where our validation necessitates comparison with single subject studies.

## Validation

Validation of any neuroimaging methods is problematic because it is not possible to directly measure brain activity at every location in the brain. Nevertheless, three methods are obvious candidates for assessment of SPECTRE’s validity.

The first is comparison with functional MRI (fMRI), the current method of choice for whole brain spatial localization of brain activity. However, association of fMRI with a “standard” for EEG is problematic because it is not measuring electrical activity, but the magnetization changes in hemoglobin as blood becomes deoxygenated during brain activity. The timescale and location of these changes can be vastly different than those produced by EEG signals. Nevertheless, its capability of spatially localizing activated brain regions merits a comparison. The most direct comparison is between fMRI and EEG data collected simultaneously, which guarantees that the brain activity measured is identical in both experiments. Such “simultaneous fMRI/EEG” experiments are not particularly common as collecting EEG data within an MRI scanner during imaging is notoriously difficult, and the MR imaging procedure significantly distorts the EEG signal. However, a recent open-source study provides such data which is sufficient for our purposes.

A more direct method for validating the ability of SPECTRE to reconstruct localized electrical activity can be constructed from intra-cranial EEG (iEEG) recordings collected during epilepsy studies. Such measurements consist of specially designed EEG sensors distributed linearly along a probe that is inserted deep within a brain that has been exposed by surgical removal of a portion of the skull. By selecting only these electrodes near the brain surface from the full array of electrodes, we can synthesize an artificial surface distribution of electrodes to mimic a standard non-invasive EEG experiment (albeit with a limited coverage of the brain). We have access to such data through an ongoing study which enabled this method of validation as well.

Lastly, a comparison with current “source localization” methods would seem to be in order [39]. This comparison turns out to be the most problematic as these methods all employ a very different, and quite limited, physical model for the EEG signal, and suffer from computational limitations as well. Despite attempts to make a reasonably valid comparison, it was determined that this was not possible, as described below.

### Validation with simultaneous fMRI/EEG visual task

It is notoriously difficult to get high quality EEG data in simultaneous fMRI/EEG studies as the presence of the rapidly varying magnetic fields present in an fMRI acquisition distort the EEG signal. However, one recent open-source simultaneous fMRI/EEG study of a well controlled visual task (the periodic flashing checkerboard) on multiple subjects ([40], available from the Nathan Kline Institute) provides important data to address this question.

**Figure 1:**
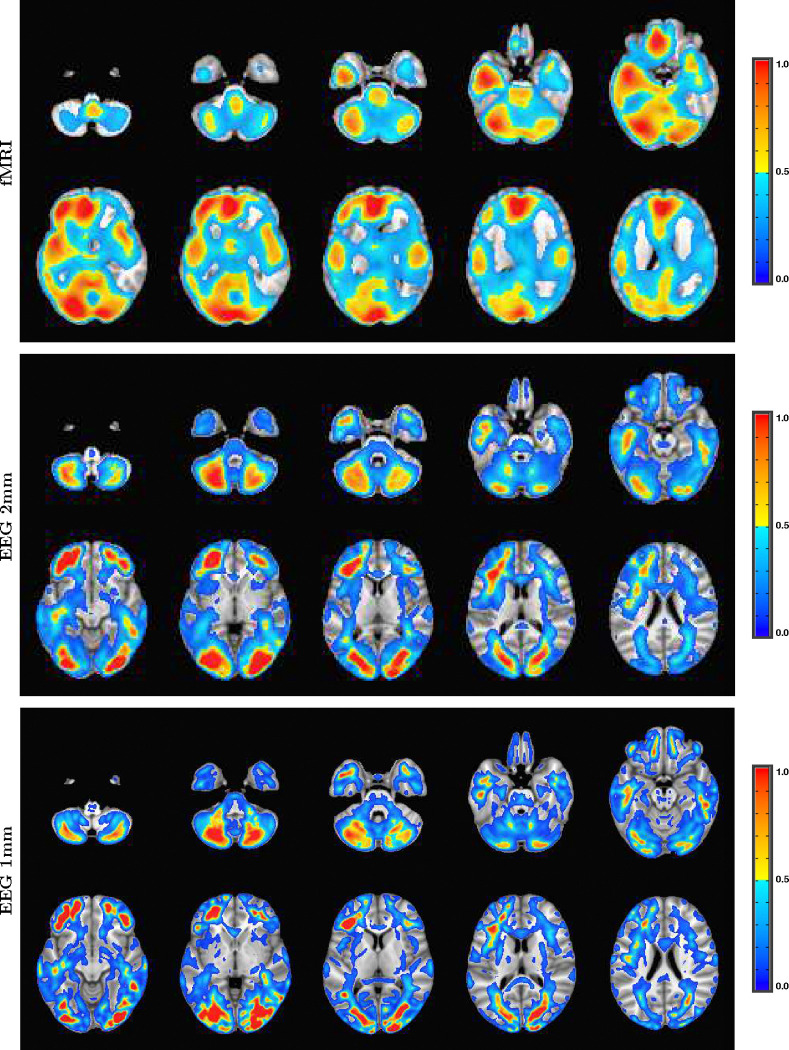
Comparison of EFD reconstructed fMRI activity (top) with SPECTRE EEG reconstruction in the frequency band 0-1hz at both 2mm (middle) and 1mm (bottom) spatial resolution (axial view) from a single representative subject from an open-source study with simultaneous fMRI and EEG [40]. In both cases, the weighted sum of the power over all modes is shown. The task was a simple 8Hz flashing checkerboard with 4 on/off cycles. The non-linear registration of the fMRI to the anatomical template in the fMRI data (top) is imperfect because of significant field-induced non-linear geometric distortions in the fMRI data. The colors are the weighted sum over all estimated amplitudes of the activation modes. Intensities are scaled between 0 and 1, and thresheld at .6.

The fMRI procedure samples the data at relatively course temporal sampling and thus is most sensitive to low frequency variations in BOLD activity. The most useful comparison of SPECTRE with fMRI is therefore in the lowest frequency band, 0−1*Hz* (for details on the fMRI acquisitions, see Appendix Appendix B). The SPECTRE reconstruction in this frequency band is shown in Fig.1 and demonstrates the ability of SPECTRE to faithfully reconstruct the spatial distribution similar to fMRI. . Importantly, this comparison was performed on data from a single subject, since brain activity patterns can vary significantly between individual and averaging over multiple subjects obscures specific spatial variations important for validation. In the top rows of Fig. 1 is shown the fMRI EFD mode that automatically detects the activation in the primary visual cortex. In the middle row are shown the SPECTRE modes reconstructed using the 2mm MNI anatomical atlas, chosen because it was closest in resolution (2*mm*^3^) to the fMRI data (~ 3*mm*^3^). The very close correspondence between the spatial patterns is evident.

The bottom rows in Fig. 1 clearly demonstrate one of the most compelling, and perhaps surprising, aspects of SPECTRE - its ability to reconstruct activation at spatial resolution *significantly higher* than fMRI. This is a consequence of the SPECTRE reconstruction being based on the solution of the propagation of electromagnetic wave through specific tissue morphologies and bioelectric properties, provided by arbitrary resolution anatomical MRI data. The finer the resolution of the MRI scans, the more details can be available for the reconstruction. This is of course dependent upon the number and distribution of the EEG sensors, but certainly holds for the standard array configurations used in this paper.

**Figure 2:**
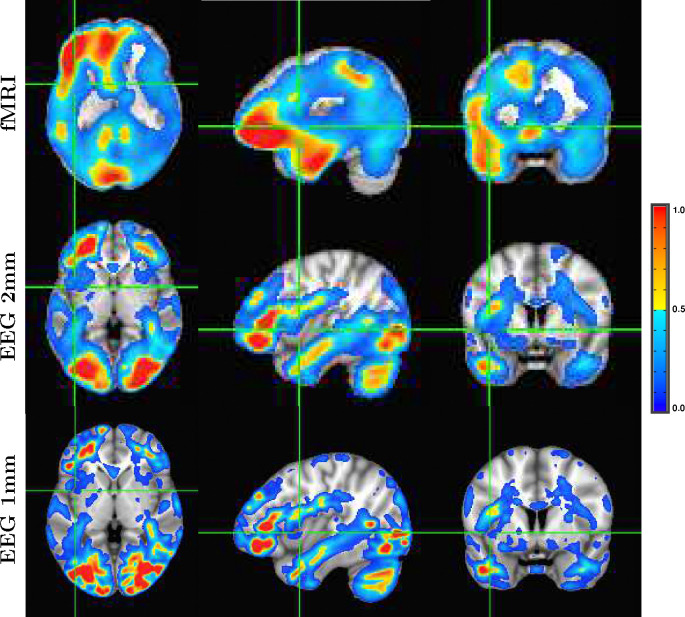
A detailed visualization of three orthogonal views of data in Fig.1) demonstrating the fine spatial resolution produced by SPECTRE, and the ability to reconstruct activations in regions prone to severe distortions in fMRI, such as the frontal lobes and cerebellum. The colors are the weighted sum over all estimated amplitudes of the activation modes. Intensities are scaled between 0 and 1, and thresheld at .6.

Although it is an almost universally believed notion that EEG and fMRI are complementary because EEG has excellent temporal resolution but poor spatial resolution, while fMRI has poor temporal resolution but good spatial resolution, in fact SPECTRE EEG reconstructions can achieve much higher *intrinsic* temporal *and* spatial resolution. Moreover, because there are no spatial distortions in SPECTRE, this mitigates one of the aspects of fMRI that most confounds spatial localization through signal loss and non-linear geometric distortions. This is shown in Fig.2.

**Figure 3:**
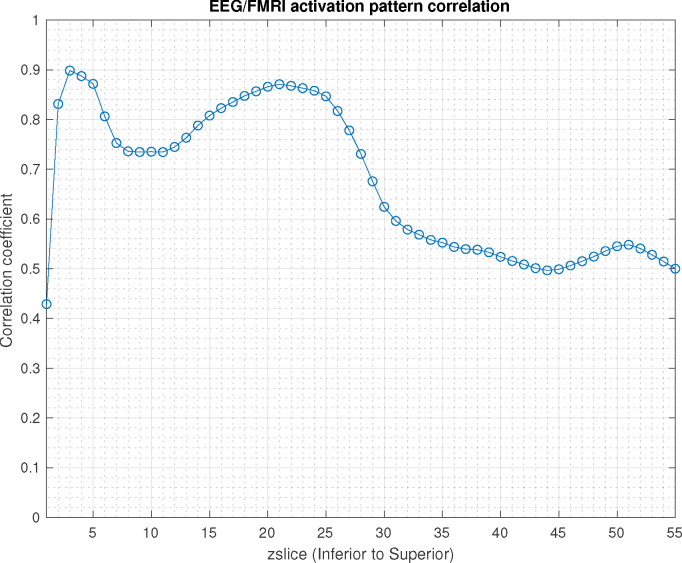
Correlation coefficient in each axial slice (from inferior to superior) between the activation patterns estimated by SPECTRE EEG and the fMRI for the data in Fig.1). Regions of high correlation indicate the similarity in activation patterns detect between the two completely different neuroimaging methods (SPECTRE and FMRI). Reduction of the correlations in the superior regions of the brain, possible due to the increased distortions in that region in this fMRI dataset.

The slice-by-slice correlation coefficient between the activation patterns estimated by SPECTRE and fMRI are shown in Fig.3. Regions of very high correlation, most notable in the inferior brain regions, indicate the similarity in activation patterns detect between the two completely different neuroimaging methods (SPECTRE and FMRI). The correlations are not as strong in the superior regions of the brain, possible due to the increased distortions in that region in this fMRI dataset. Even with perfect activation detection by both methods, the correlations would not be perfect (i.e, 1) as the two methods are measuring different physical processes. However, the smooth variations are indicative of non-random correlations between two vastly different imaging modalities.

It should be noted that the ’simple’ periodic flickering checkerboard stimulus not only activates the primary visual cortex but activates other visual and supplementary fields as well, as is evident from the activity patterns in Fig.1. A simple stimulus does not imply a simple activation pattern. This notion was a primary motivation for our development of the EFD method for fMRI [24]. The activation mode reconstructions for both the fMRI and SPECTRE data are based on the EFD which detects complex non-linear interacting spatial-temporal modes of activity [24]. Thus although the task is a ’simple’ visual stimulation, our analysis is not expected to simply detect activity in only the visual cortex, as would be produced by a more standard regression approach [40], but in a more complex set of brain networks. Indeed, multiple EFD modes are produced, though we have only shown the one incorporating the primary visual cortex. As we have argued previously [24], EFD analysis is more sensitive than simple regression techniques to the complex brain activation patterns predicted by neuroscience, and less sensitive to erroneous identification of noise or non-independent modes than the independent component analysis (ICA) [24]. Indeed, one of our observations from both the fMRI and EEG data used in this study [40] is the appearance of PFC activations associated with visual stimulation, which has been suggestive of conscious visual perception [41, 42]. Addressing this question is beyond the scope of the current paper.

### Validation with simultaneous fMRI/EEG Attention Paradigm

Simultaneous EEG/fMRI were collected from subjects within a standard clinical 3T MRI scanner (see Attention paradigm data for details). The stimuli and paradigm are described in detail in [43]. Briefly, bimodal stimuli consisting of short (~ 1*s*) streams of simple tones (600 and 1000 Hz) alternating at 10 Hz were delivered concurrently with phase-reversing (6Hz) checkerboard patterns presented at fixation. Participants were instructed to selectively attend to either the visual or auditory aspect of the bimodal stimulus and respond when the stream of stimuli in the attended modality ends.

**Figure 4:**
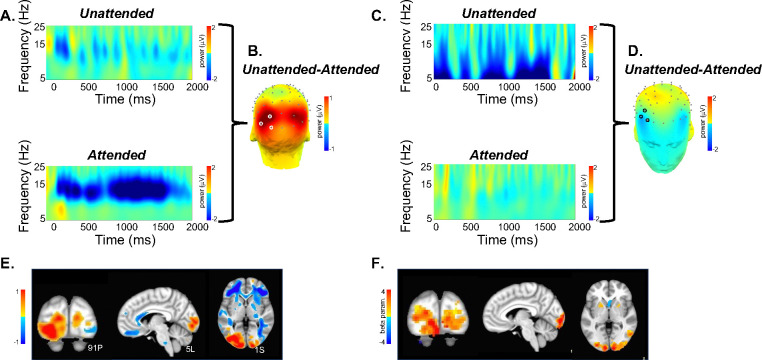
A. Baseline-corrected EEG activity from a single subject elicited by unattended (top) and attended (bottom) visual stimuli averaged across the cluster of 3 occipital electrode sites (PO7, PO3, O1) denoted in B. by white circles. Over the broad alpha frequency band (7–16Hz) there was a reduction in total power (from the pre- to post-stimulus latency interval) which was greater for attended, compared to unattended, visual stimuli. B. Scalp-topography of the mean difference in oscillatory (8–12Hz) activity for unattended minus attended visual stimuli across the 0–2000ms latency interval. As expected, attention modulated (reduced) the power of these oscillations over the visual cortex. C. As in A. for three frontal electrode sites (F6, F8, AF6) denoted in D. by black circles. In contrast to visual cortex, in bilateral frontal regions, unattended visual stimuli elicited a greater reduction of oscillatory activity between 5–10Hz (theta-alpha frequency). D. Frontal view of the unattended minus attended difference topography between 0–2000ms in the 8–12Hz frequency band. E. SPECTRE power estimates derived from mean (baseline-corrected) oscillatory power between 0–2000ms and across 8–12Hz for the same subject shown in panels A-D, superimposed on the MNI template brain. Hot colors (yellow to red) indicate greater attention-related modulation (reduction) of activity and the inverse for warm colors (light to dark blue). F. BOLD signal (beta parameter estimate) contrasting activation to visual stimuli when attended versus activation to the same stimulus when unattended. Attention-related enhancement of the BOLD signal in visual cortex mirrors the reduction in alpha power obtained in the same subject using EEG.

**Figure 5:**
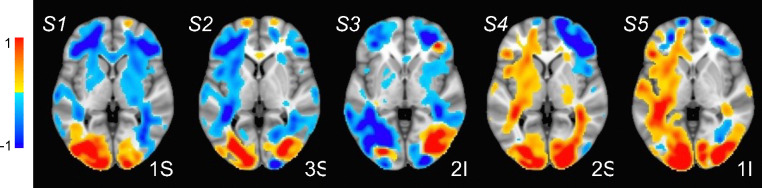
Estimated localization of neural activity for 8–12Hz oscillatory activity (unattended minus attended; 0–2000ms) for five participants (S1-S5). Colors are as in 1E. A prominent bilateral occipital source associated with increased attentional modulation is observable in all participants. A bilateral source localized in middle frontal cortex and indicating less modulation is also consistently observed across participants. Note that these are difference maps from the weighted sum over all estimated amplitudes of the activation modes, so that the intensities are scaled between −1 and 1, and thresheld at absolute value .6.

SPECTRE processing was performed in the alpha band. The appearance of visual stimuli elicited a reduction of ongoing alpha (7–14Hz) activity (“event-related desynchronization”, ERD) over occipital cortex, believed to occur when cortical regions are brought “on-line” for information processing [44]. As in previous studies, e.g. [45], attended visual stimuli elicited increased (more negative) amplitude of the alpha ERD compared to unattended stimuli (Fig. 4A,B). In contrast, unattended, compared to attended, visual stimuli elicited a greater reduction in ongoing spectral activity within the 5–15Hz frequency range over bilateral middle frontal cortex (Fig. 4C,D). We estimated the neural sources of these attention-related modulations of oscillatory activity across the 8–12Hz frequency band which encompassed both the occipital and frontal activities (Fig.4E). Their anatomical localization was remarkably consistent across several individuals (Fig.5).

**Figure 6:**
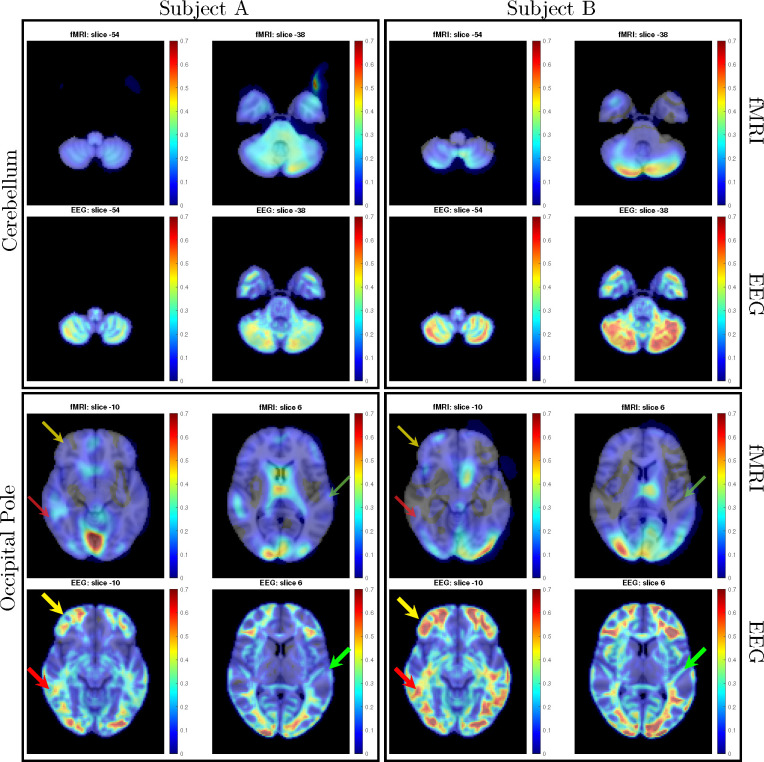
Direct comparison of activation maps from two participants (Subject A, left; Subject B, right) in the bimodal (auditory + visual) stimulation paradigm described for Figures 3 and 4. In each subject, two brain regions – the cerebellum and the occipital pole (top and bottom rows, respectively), were delineated based on the MNI atlas and EFD activation maps were correlated across these entire regions. Correlation coefficients were as follows: for Subject A, cerebellum=0.74, occipital pole=0.70; for Subject B, cerebellum=0.70, occipital pole=0.84. Correlations were computed only for regions exhibiting activation levels above 0.1. In contrast to fMRI, the SPECTRE technique identified robust activations in bilateral middle and inferior frontal cortex (indicated by yellow arrows) and middle temporal cortex (red arrows). It also discerned activations along the superior temporal cortex, including areas encompassing the primary auditory cortex (green arrows).

A direct comparison of the activation maps derived from both fMRI and EEG using SPECTRE for single study within two subjects (i.e. without any average over studies or subjects) is shown in Fig.6. The comparison is made by choosing specific regions of interest defined in the MNI atlas (occipital cortex and cerebellum) and correlating the activation maps derived from EFD for fMRI and SPECTRE from EEG. Comparison of the similarity of activated regions in individual subjects is generally a non-trivial problem. This is particularly true in the current case where the spatial distortions in fMRI (and lack of them in SPECTRE) make measures such as mean-squared error difficult to interpret. Therefore computation of the correlation coefficient over a predefined atlas ROI is a reasonable conservative measure of statistical significance.

**Figure 7:**
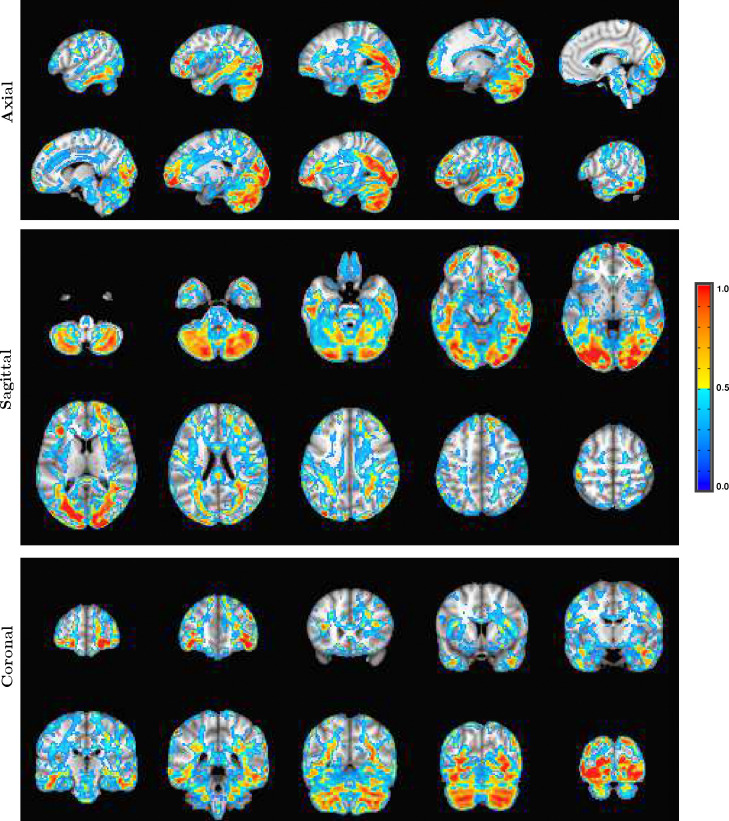
Orthogonal slices from whole brain electric field activation maps from a 2mm SPECTRE reconstruction of EEG data from a single subject in the attention study. The colors are the weighted sum over all estimated amplitudes of the activation modes.

As an example of the type of whole brain electric field activation maps that are possible with SPECTRE is shown in the montage of orthogonal slices from a 2mm reconstruction Fig. 7 from one of the subjects of this same attention study.

#### Statistical Significance of Simultaneous EEG/fMRI results.

Direct comparison of activation maps from two participants in the bimodal (auditory + visual) stimulation paradigm described for Figures 3 and 4 is shown in Fig.6. In each subject, two brain regions – the cerebellum and the occipital pole (top and bottom rows, respectively), were delineated based on the MNI atlas and EFD activation maps were correlated across these entire regions. Correlation coefficients were as follows: for Subject A, cerebellum=0.74, occipital pole=0.70; for Subject B, cerebellum=0.70, occipital pole=0.84. Correlations were computed only for regions exhibiting activation levels above 0.1. In contrast to fMRI, the SPECTRE technique identified robust activations in bilateral middle and inferior frontal cortex (indicated by yellow arrows) and middle temporal cortex (red arrows). It also discerned activations along the superior temporal cortex, including areas encompassing the primary auditory cortex (green arrows). We emphasize that the difference in activation maps provided SPECTRE and and fMRI are not expected to be identical, as EEG and fMRI are not measuring the same physical quantitites. Indeed fMRI is measuring rather poor proxies of the brain electrical fields. Therefore it would be remarkable if these two methods did not have significant differences. Fig.4 provides the best example, where our EEG method showing deactivation (blue in E) is consistent with what can be considered a Gold Standard for EEG - the direct surface recordings near the scalp where the deactivation in the frontal lobes (C,D in blue) corresponds. It should also be noted that this is not simply a visual task, but an attention task, for which these activation patterns are well known, and thus provides yet another form of validation.

Therefore, the high correlation coefficients between the maps Fig.6 are therefore indicative of the consistency between the fMRI and SPECTRE results in the ROI. Note that this does *not* imply similarity over the entire region shown. Indeed, the SPECTRE results show enhanced sensivity to activation in regions not seen in the fMRI.

### Validation through Intra-Cranial EEG recordings: Surface electrode vs complete electrode array

**Figure 8:**
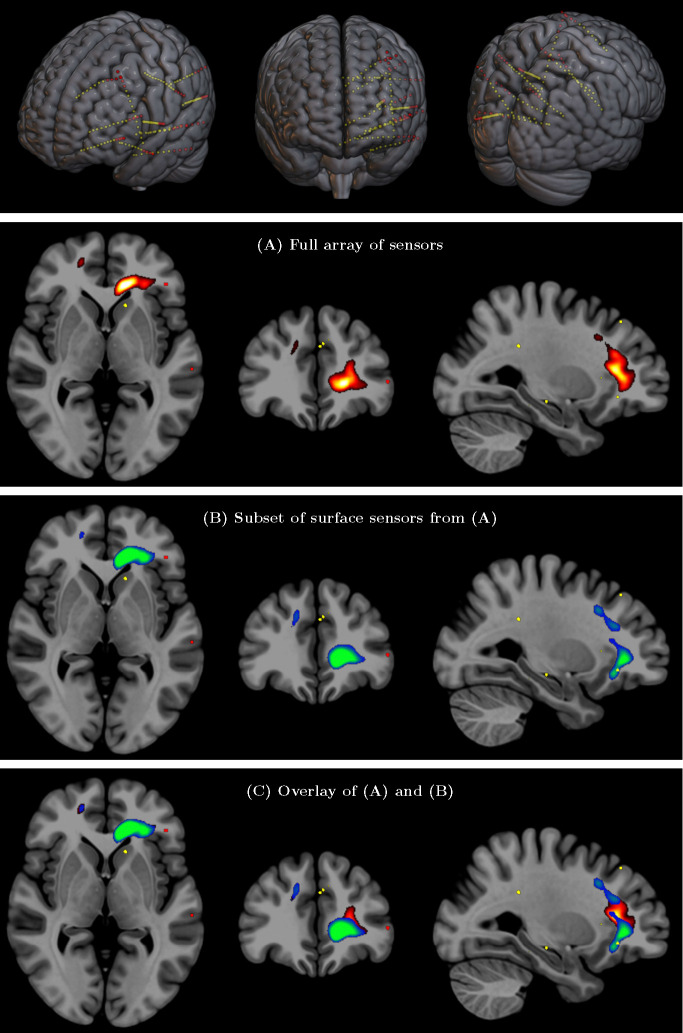
(Top row) Full array of intra-cranial EEG contacts from a recording in a medically refractory epilepsy patient (yellow dots). Red dots indicate subset of surface-only electrodes to mimic a standard non-invasive (i.e., extra-cranial) EEG study. SPECTRE α band reconstruction from (A) full array of intra-cranial EEG sensors from an epilepsy study (yellow dots) in top row and (B) from subset of surface electrodes (red dots) in top figure. (C) Overlay of (A) and (B) validating that the surface based is correctly reconstructing the local electric field potential detected by the intra-cranial electrodes.

**Figure B.17:**
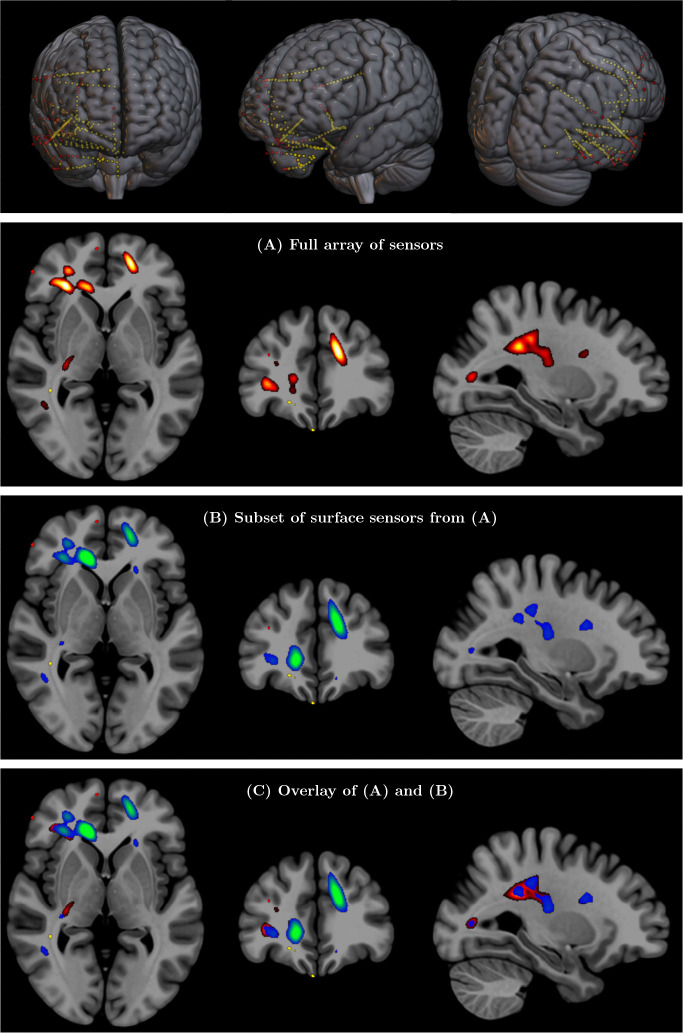
Validation of SPECTRE with iEEG data for subject 2 (see Fig.8 for details.)

While comparison with fMRI can validate the correct detection of activated brain regions and networks, as shown in the previous section, it cannot inform the question of correct detection of electrical signals, since fMRI is based on a completely different contrast mechanism related to blood oxygenation. A direct validation of SPECTRE’s ability to faithfully reconstruct deep electromagnetic activity is, to our knowledge, only achievable with one type of data: intra-cranial EEG (iEEG) recordings such as those used in medically refractory epilepsy patients for seizure onset localization where the electrodes are known to be adjacent to the site of electrical activity [46, 47]. We analyzed an iEEG recording of a seizure localized in the left mesial temporal region acquired at Northwell Health, NY. All implanted electrodes are shown in Fig. 8 (Top row) with each yellow dot depicting one recording contact. Comparing the SPECTRE reconstruction using all of the sensor data with one using only a subset of the data comprised of only the sensors on the surface of the brain (red dots in Fig. 8 (Top row)) allows the quantitative assessment of how closely the results from a set of surface electrodes correspond to those produced by intra-cranial measurements recording signal very close to the sources. The results are shown for the alpha frequency band in Fig. 8 and reveal a very close correspondance between the SPECTRE mode reconstruction. Results for subjects 2–4 are shown in the Figs.B.17 to B.19 in the Supplementary Material and show similar agreement.

### Validation through Intra-Cranial EEG recordings: Comparison with Simulation

A traditional approach for validating estimation methods is to compare results against a "ground truth" derived from numerically simulated signals. This is a standard procedure in source reconstruction methods, where simulated point dipole sources are embedded within a brain model (e.g., a high-resolution MRI scan). The forward problem is then solved to generate the dipole fields at the brain surface, and the resulting simulated signals are used to estimate the original source locations. While this approach works well for dipole-based models of brain activity, it is not directly applicable to the more realistic WETCOW model of brain waves.

However, an alternative validation strategy is possible by comparing SPECTRE data to the most reliable ground-truth data available: intracranial EEG (iEEG). One of the most striking predictions of the WETCOW theory is the presence of coherent, sustained cortical wave loops—a phenomenon demonstrated through numerical simulations in a realistic brain model derived from high-resolution anatomical (HRA) data (Fig. 10, Top). This prediction provides a natural benchmark for validating the SPECTRE method.

An application of SPECTRE to a WETCOW analysis of iEEG recording of several epileptic seizure onsets in insular posterior opercular and in hippocampus areas that provides our first experimental evidence of the existence of our hypothesized cortical loops is shown in (Fig.10 (bottom right), SPECTRE reconstruction confirms the existence of cortical wave loops Fig. 10, Bottom right), consistent with the numerical simulations shown in Fig.10 (top). The intracranial leads are shown in Fig.10 (bottom left).

#### Statistical Significance of Intra-cranial EEG (iEEG) results.

**Figure 9:**
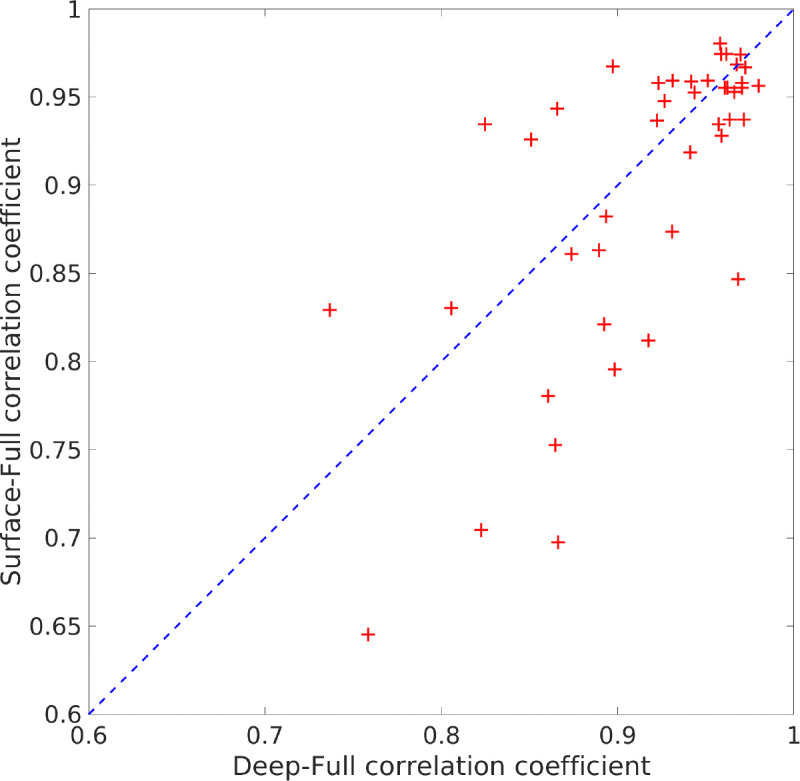
Statistical comparison of full vs surface intra-cranial EEG estimates. The horizontal axis are the correlation coefficients between the estimates obtain the full set of electrodes and the deep electrodes (adjacent to the source). The vertical axis are the correlation coefficients between the full set of electrodes and just the surface electrodes, as would be collected in a standard (extra-cranial) EEG experiment. The results are highly correlated and thus support the claim that the SPECTRE recontruction of the spatial distribution of deep electrical activity from the surface measurements accurately reflects the true spatial localization of the deep electric fields.

**Figure 10:**
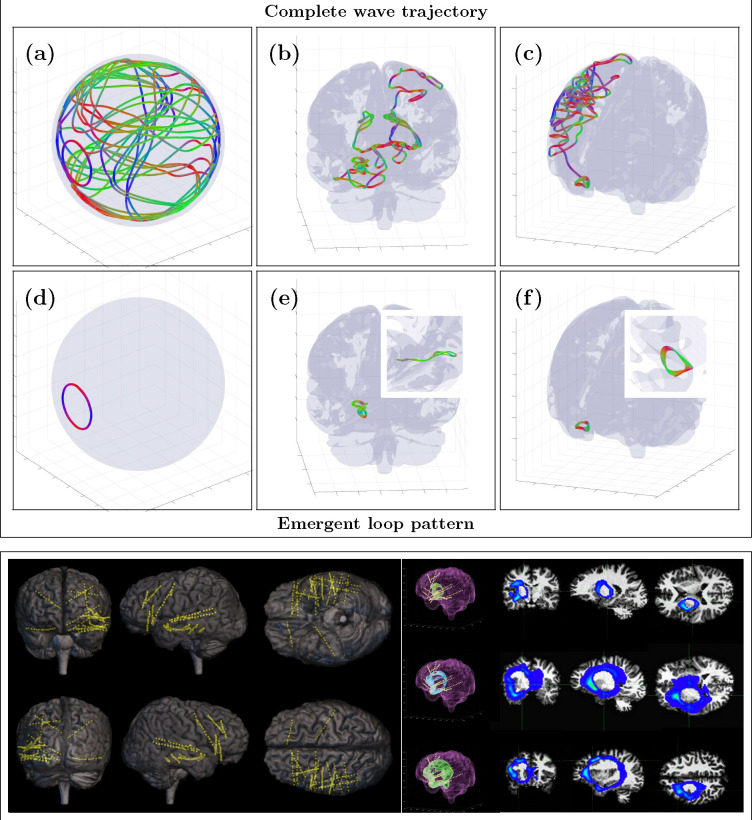
Validation of WETCOW model with intra-cranial measurements and SPECTRE reconstruction. (Top) Examples of wave trajectories obtained in simulation of wave propagation in real data cortical fold tissue model. Panels (a)-(c) show the complete trajectories and panels (d)-(e) show the emergent stable wave loops. The spherical cortex shell model of is used for panels (a) and (d) and the cortical fold model is used for panels (b),(c),(e), and (f) (reprinted from [9]). The colors encode wave propagation: red - left/right, green - anterior/posterior and blue - dorsal/ventral. (Bottom) (left) EEG contacts and (right) detected WETCOW cortical loops from iEEG recordings of epileptic seizure onset in insular posterior opercular area

A deep-surface-full comparison of modes for seizures datasets was run for all 5 iEEG subjects with 44 events total. The correlation plot shown in Fig. 9 demonstrates that the correlations are very high. We ran 3 t-tests on Fisher’s Z-transformed correlation values (full-deep/full-surface, full-deep/deep-surface, full-surface/deep-surface) and the t-tests show that full-deep/full-surface correlations are very similar (null-hypothesis is not rejected, p=0.2368, t(43)=1.19, p>.001,95%, 95% CI [−0.0370, 0.1457], SD = 0.3004), but the full-deep/deep-surface and full-surface/deep-surface t-tests show statistically significant differences (p=1e-8, t(43)=7.05, p>.001, 95% CI [0.1827, 0.3291], SD = 0.2408, and p=8e-6, t(43)=5.06, p>.001, 95% CI [0.1213, 0.2818], SD = 0.2640). These results support the claim that the SPECTRE recontruction of the spatial distribution of deep electrical activity from the surface measurements accurately reflects the true spatial localization of the deep electric fields.

### Investigation of the ’reward circuit’

Having validated the SPECTRE method directly with simultaneous fMRI/EEG, iEEG, and an attention paradigm, we investigated the ability of SPECTRE to faithfully reconstruct the well known neural ’reward circuit’ that is one of the most important in understanding human cognition, emotion, and behavior [48–50] and is of great clinical significance in the understanding of addiction [51, 52], mood disorders [53, 54], and a variety of other conditions [55].

We demonstrate that SPECTRE using standard EEG data can accurately map human reward pathways akin to results previously only seen via fMRI. Indeed, fMRI results have highlighted a reward system within the brain that includes midbrain dopamine producing regions (the substansia nigra pars compacta, the ventral tegmental area), the ventral striatum, and multiple regions within the human prefrontal cortex [56]. Other research using fMRI and source localization of EEG data suggests that the anterior cingulate cortex also plays a key role in reward processing [57]. In a unifying theory, it has been proposed that all the aforementioned regions work together as a neural system for the optimization of reward driven behavioral change (i.e., reinforcement leaning; [58].)

This is of particular clinical significance because addictive behaviors have long been known to be subserved by specific brain regions operating in concert as the reward circuit [59–63]. The reward circuit is involved in processing rewarding stimuli of any sort and in drug addiction, substances of abuse (e.g., amphetamine) increase dopamine release in a protracted and less regulated manner as compared to typical stimuli, resulting in synaptic plasticity, and altered functioning of this circuit over time.

For our analysis we used large gambling task dataset that includes 500 participants available for download from www.osf.io/65x4v/. The details of the dataset and an extensive analysis using standard EEG analysis methodologies are presented in [64]. The relevant information from this study is presented in Supplementary Materials Reward circuit data.

**Figure 11:**
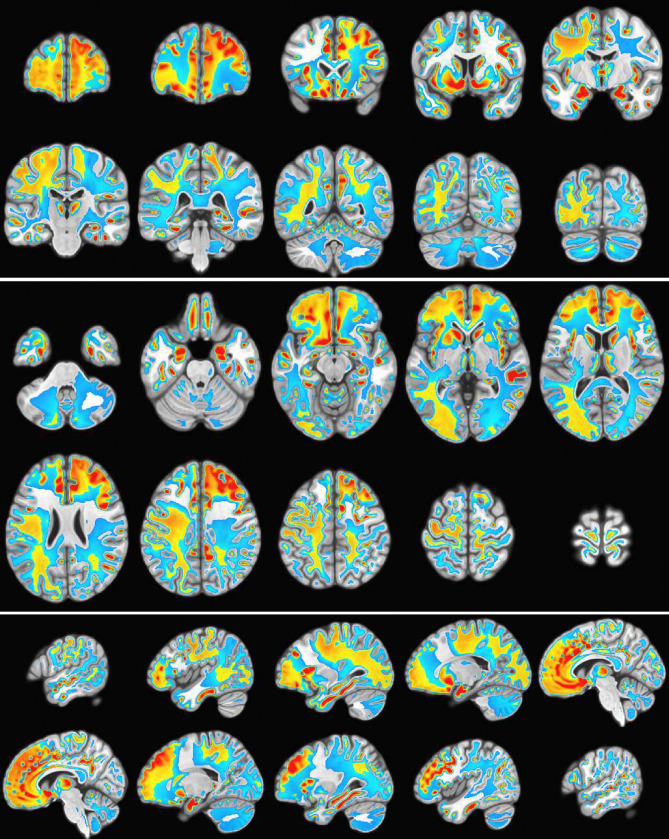
Gambling task EEG from 500 subject cohort. Alpha power of the weighted summed over the first n=10 SPECTRE modes . Activation in key regions of the reward circuit, including the frontal lobes, paracingulate gyrus, accumbens, and amygdala are clearly evident. Negative activation (i.e., deactivation) is evident in the supplementary motor cortex and the left temporal-parietal regions.

For each subject trial n=10 power modes were calculated and summed to form the single space-time SPECTRE mode (see Mode reconstruction). Fig. 11 shows three orthogonal slices of the difference in EFD power summed over all modes between conditions, averaged over all subjects. Activation in key regions of the reward circuit, including the frontal lobes, anterior cingulate gyrus, accumbens, and amygdala are clearly evident. Strong negative activation (i.e., deactivation) is evident in several structures, including the supplementary motor cortex, and the parietal operculum cortex. Activation is also apparent in the lingual gyrus and around the calcarine fissure and, as expected, in bilateral subcortical structures.

**Figure 12:**
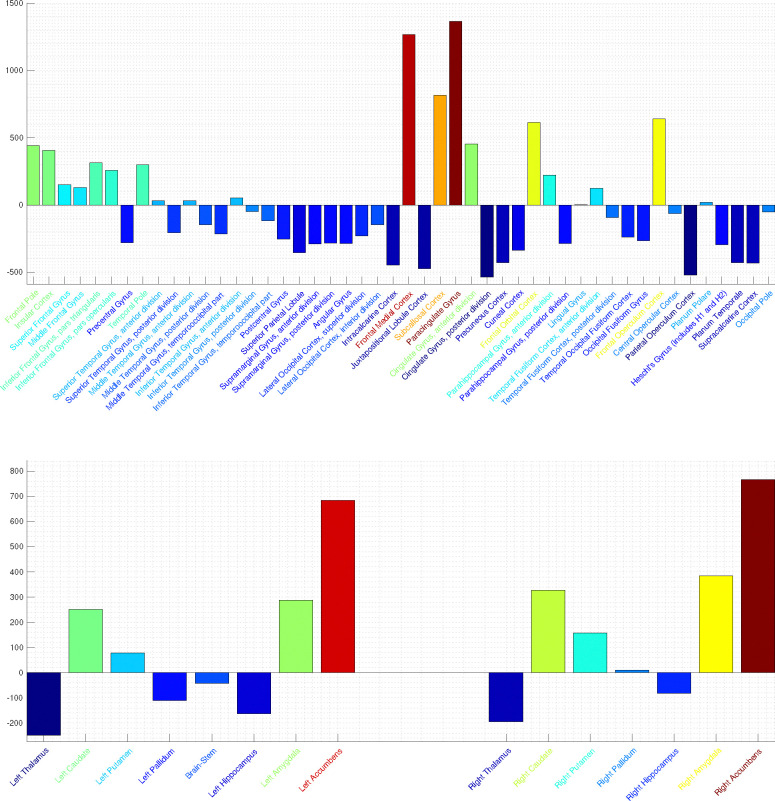
SPECTRE power per brain region in the Harvard-Oxford 2mm cortical (top) and subcortical (bottom) atlases. Colormap is from hot/yellow (activated) to blue (de-activated). Activation in key regions of the reward circuit, including the frontal lobes, paracingulate gyrus, subcallosal cortex/nucleus accumbens, and amygdala are clearly evident. Negative activation (i.e., deactivation) is evident in the supplementary motor area, posterior cingulate, and thalamus. Activation of the important reward element accumbens is evident in the bottom plot. Also of note is the relatively similar activation in the bilateral subcortical elements.

In Fig.12 is shown the power per brain regions as defined by the Harvard-Oxford 2mm cortical (top) and subcortical (bottom) atlases. In the cortical regions (top), strong activation is apparent in the frontal cortex (medial, orbital, operculum), cingulate gyrus, paracingulate gyrus, and unsular cortex. Activation in the accumbens is apparent from the data in the sub-cortical atlas Fig. 12 (bottom). These activated regions are consistent with the known elements of the human brain reward circuitry.

**Figure 13:**
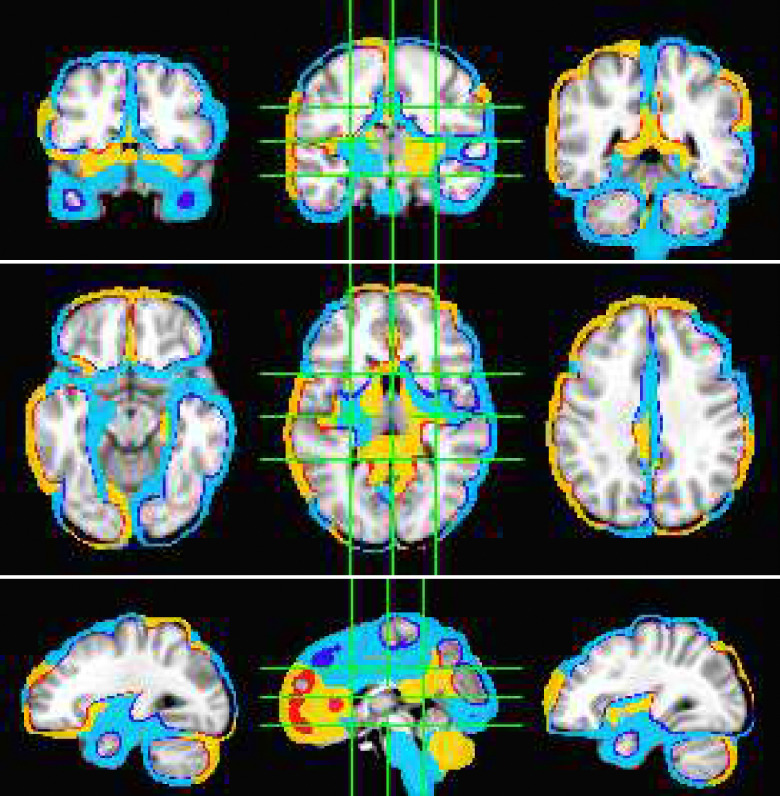
Statistical significance. t-statistic between the SPECTRE power modes pre- and post-stimulus reward experiment. Calculations were performed using the standard AFNI 3dttest++ algorithm. Yellow/red color reflects positive changes, blue color reflects negative changes. Significance threshold was $p=10^{-8}$, indicating strong statistical significance.

Images of statistical significance (p<.0001) are shown in Fig.13. It should be noted that the determination of statistical significance with SPECTRE by ’traditional’ methods is potentially misleading as they will tend to *underestimate* activation significance. The estimation of the modes in SPECTRE employs EFD [23, 24] which is a probabilistic formulation that *by construction* incorporates space-time neighborhood connectivity so that spatially and temporally coherent patterns (“clusters”) are more probable. Traditional methods have the option for “clustering” regions of activation *post-hoc* into their general class of techniques called “bootstrapping” or “permutation inference”. Cluster post-detection of an activation is incommensurate with our view of the estimation process wherein the clustering in space-time is a key component indicator of high-probability regions of space-time. Spatially and temporally coherent patterns maybe of low amplitude with apparent low significance by traditional means, but those intensities are within a mode that contains very high significance in cortical regions (e.g., Fig.13) which is predicted by the WETCOW model.

#### Statistical Significance of Reward circuit results.

Mass univariate voxel-wise statistical analysis across the whole brain was performed using AFNI 3dttest++. The first level fixed effects were analyzed to produce contrast estimates computing the mean activation for each condition (the SPECTRE power modes obtained for pre- and post-stimulus reward experiment). Statistical significance (t-statistic) between the SPECTRE power modes pre- and post-stimulus reward experiment. Calculations of statistical significance (two sample t-statistic) between the SPECTRE power modes pre- and post-stimulus reward experiment were performed using the standard AFNI 3dttest++ algorithm. Significance threshold was p=1e-8, indicating strong statistical significance. Permutation/randomization multiple comparisons correction method to control the family-wise error rate (FWE) and false discovery rate (FDR) was used with AFNI’s 3dttest++ cluster-level thresholding through the -ClustSim option of AFNI 3dttest++ algorithm. Images of statistical significance (p<.0001) are shown in Fig. 13. These results support the claim that SPECTRE can reliably reconstruct whole brain electric field activity.

### Comparison with state-of-the-art Source Localization methods

There is a long history of attempts to spatially localize EEG activity and these are generally called “source localization” or “source reconstruction” methods [65–68]. These methods are fundamentally different from the SPECTRE approach as they are solving a different problem than SPECTRE (as described in Section 2) that involves numerous stringent assumptions about brain electrical activity such as a fixed set of static dipole sources, an idealized geometric model of the head reduced to a few (typically 3) shells, that spatially close points are more likely synchronized, and the smoothness of the solution. (see [69] and references therein).

These methods all implicitly assume the “quasi-static” approximation to the EM field equation which entails ignoring the time dependent terms in Maxwell’s equations, which are dependent on tissue conductivity properties which are themselves frequency dependent. The resulting solutions are therefore static, have no frequency dependence, and are insensitive to the detailed spatially variable electrical properties of the tissues. However, as discussed in detail in the development of the WETCOW model [8–10], these assumptions are incompatible with the basic physics of brain electrical activity. The SPECTRE approach is to employ the WETCOW model and solve the actual physical problem of the complete Maxwell’s equations in an inhomogeneous and anisotropic medium. It is specifically these dependencies that give rise to the previously undiscovered WETCOW waves that propagate preferentially along the gradients of local tissue inhomogeneity and anisotropy and thus propagate preferentially *perpendicular* to neuronal pathways. The WETCOW theory provides a comprehensive framework for characterizing the propagation of EM fields through the complex brain tissue microstructure and larger scale morphology (e.g., cortical folding), and provides the dynamic solution to the electric potential field necessary to solve the EEG inverse problem.

The problem of spatially localizating the EEG signal involves estimating the most probable distribution of electric field amplitudes given an array of sensors. This is essentially a problem of correctly modeling the physics of how electromagnetic waves propagate through the complex environment of a the convoluted brain tissue morphology and the anisotropic and inhomogeneous nature of brain tissue. The current state-of-the-art approach to this problem, called ’source localization’ such as *low resolution electromagnetic tomography* or LORETA algorithm with its many variations [65–68], also called ’EEG source imaging’ [70, 71]) involves using a pre-defined brain atlas, arbitrarily placing dipole source on the surface, and calculating the contribution from these sources. Some methods propose using fMRI as a prior, which has the disadvantage of requiring fMRI acquisitions [72–75]. The current source localization methods are based on a static model for the electric field caused by a fixed set of pre-defined dipole sources. (see [2] for a review of current methods). This model is inherently limited because in reality the brain’s electrical field variations are time dependent and generated by an essentially continuous distribution of sources throught the entire brain. This description is the essence of the WETCOW theory [8–10] which describes how highly coherent localized electric field phenomena, such as cortical wave loops and synchronized spiking, are produced by the complex non-linear interactions of waves across multiple spatial and temporal scales.

The theoretical discussion above in Section 2 clarifies that the problem being solved by SPECTRE is the inversion of a dynamical wave equation model in contrast to the solution of Poisson’s equation for static sources being solved by traditional ’source localization’ methods. Therefore is makes little sense to compare the two methods, as they are attempting to solve two completely different problems. However, it perhaps serves some purpose to illustrate with a specific practical example a most basic computational problem encountered if one tries to even attempt a ’source localization’ solution with the bases state even remotely resembling the setup used in SPECTRE.

In a typical application of the SPECTRE method, we use an MNI volumetric grid with 2mm (902629 voxels), 1mm (7221032 voxels), or 0.73mm (11393280 voxels). All voxels in our models are considered sites of electromagnetic activity consistent with the local intravoxel tissue characteristics (via (13)) rather than any assumed dipolar form used in source localization methods. This setup is facilitated by the pseudo-spectral computational approach used in SPECTRE detailed above in Numerical Implementation. Our highest resolution (0.73mm) SPECTRE processing can be completed on a modern workstation using 16–20Gb of memory in a matter of minutes.

For comparison with a current state-of-the-art source localization method, we downloaded the currently available LoretaKey1 software [68], which uses as a default a set of 6239 fixed dipoles. In order to make a fair comparison, we tried to use LoretaKey1 with a number of dipoles comparable to the number of voxels we use for our lower resolution (2*mm* voxel) reconstruction in this paper. We began with the equivalent 902629 voxels used by SPECTRE in the 2*mm* reconstruction, but found that LoretaKey1 is not able to handle this size because of memory limitations (i.e., “outof-memory” crashes). We subsequently scaled down the number of dipoles in by factors of 2, 4, 10, and 20 times. Only with around 45K dipoles were we able to make Loreta run. It ran for approximately 24 hours, but then again crashed due to out-of-memory problems. And it should be kept in mind that this is in the absense of even any attempt by Loreta to provide a solution to a time-depedent wave equation. On the contrary, our processing with 2mm, requires around 650Mb of memory and takes only a matter of minutes to complete. At this point it was decided that it was not possible for Loreta to provide a result that would usefully inform the efficacy of the SPECTRE method.

## Discussion

### Neuroimaging and brain activity models

The ultimate goal of functional neuroimaging is to non-invasively detect and quantify the spatial and temporal variations in brain activity in terms of functional modes or ’networks’. This requires the development of models for brain activity for the quantities being measured by the imaging modality and a reconstruction method to estimate the parameters of the physical model from that modalities data.

The two methodologies that have emerged as the modalities of choice, fMRI and EEG, offer an interesting perspective on this general problem. The recognized importance of spatially localizing brain activity led to the development of fMRI in order to leverage the ability of MRI to spatial localize anatomical regions of the brain. The price paid is that the brain activity measured was constrained to be related to the physical effects that MRI was sensitive to, which turned out to be the local magnetic field perturbations produced by the susceptibility variations due to the changes in the oxygenation state of hemoglobin [76]. The actual spatiotemporal effects measured in an fMRI experiment are a complicated combination of this effect filtered through the simulateneous influences of metabolic, blood flow, and biomechanical factors [77]. The connection of the fMRI to brain electrical activity is therefore quite indirect.

The problem faced by EEG is, in some sense, the opposite of fMRI. It directly measures the electrical activity of the brain, but does so using only measurements made by an array of electrodes placed on the surface of the head. There is no direct spatial localization capabilities with EEG. It is important to clarify what is meant by that statement. The ability of MRI to spatially localize signals from the brain is based on a physical model of how the signal is related to the location. In essence, this boils done to the Larmor expression ω(x)=γB(x), whose very simplicity belies the extraordinary history of quantum mechanics. MRI leverages this expression with an equally impressive history of engineering physics and computational science to produce modern scanners and sophisticated acquisition and analysis methods for reconstrucing volumetric data. With this viewpoint, the limitations of EEG can be seen as the absence of an appropriate physical model for the generation and propagation of brain electrical signals on which to base a reconstruction, or “inversion”, to produce images. Previous models have invoked a “quasi-static approximation” [3] that precludes the existence of a more realistic dynamical brain wave model and limit what information can be extracted from EEG data. Our recently developed WETCOW model of brain electrodynamics derived from first principles revealed the existence of measureable brain waves that can permeate throughout the entire brain volume, not just along neuronal pathways. The model depends on the detailed morphology and tissue composition of an individual’s brain. The SPECTRE method then leveraged this theory to develop a general method for reconstructing the modes of spatiotemporal brain electrical activity using a variety of additional estimation tools we have developed and along with tissue and morphology information provided by high resolution MRI anatomical data. The result is a practical, numerically efficient, subject specific method for directly reconstructing the time-depedent electrical activity throughout the entire brain volume directly from EEG measurements acquired by standard extant EEG systems.

### Comparison with functional MRI

Functional MRI (fMRI) has become the *de facto* neuroimaging method for spatial and temporal localization of brain activity. The contrast mechanism that forms the basis of fMRI is the blood oxygenation level dependent (BOLD) variations in the magnetic state of hemoglobin and its influence on the local MRI signal as a function of the local metabolism and hemodynamics [77]. Consequently, the spatial and temporal characteristics of the fMRI signal are related to blood flow and metabolic dynamics, rather than direct measures of electrical activity. In particular, the signal variations will be spatially localized in vascular pathways and the temporal variations, being related to blood flow effects, are very slow compared to electrical activity. In short, the spatial-temporal dynamics measured by fMRI need not (and, in fact, will not) correspond exactly to the spatial-temporal patterns of electrical activity. Numerous experimental realities also make fMRI problematic as a gold standard. In particular, fMRI is facilitated by enhancing the sensitivity of MRI to the BOLD contrast mechanism, which requires enhancing the sensitivity to local magnetic field variations through the use of $T_{2}$-weighted pulse sequences [78] which lead to increased geometric distortions, compromising not only spatial resolution but confounding the spatial localization of the activity in a complex, non-linear fashion. Gross distortions can lead to significantly reduced signal-to-noise and even completely unrecoverable signal loss, particularly in regions near air/tissue interfaces, such as in the prefrontal cortex (PFC). Moreover, the complex non-linear interactions between the magnetic fields and physiological variations such as respiration and cardiac pulsations produce a variety of complex spatiotemporal signal distortions [79]. While mitigating these artifacts is an area of very active research, they remain a serious problem for fMRI.

Nevertheless, certain very simple task-based fMRI experimental paradigms, such as finger tapping or rapidly flickering checkerboard stimuli, repeated at periodic on/off intervals, have been established as experiments that produce repeatable robust activations in known brain networks, and are commonly used as basic testbeds for assessment of analysis algorithms. When combined with simultaneous EEG acquisition, such experiments provide two different types of data that can be compared as a form of validation, with the proviso that these two methods are imaging different physical quantities.

While the advantages of SPECTRE over fMRI in temporal resolution are clear, what is perhaps surprising is its advantages in *spatial* resolution. The inverse solution that estimates the electric field potential from the EEG data is based on a physical model of wave propagation from tissues whose composition and geometry are derived from high resolution anatomical MRI data. The final resolution of the SPECTRE electric field modes is that of the anatomical data which is typically significantly higher (~ .5 − 1*mm*)) than the resolution of an fMRI image (~ 2*mm*). (There are, of course, limitations depending on the number of electrodes in the EEG system.)

But it is also important to recognize that the question of resolution in fMRI is not just a question of the prescribed image resolution of the acquisition. The BOLD physical mechanism that generates the fMRI contrast is a subtle variation in the magnetic susceptibility which causes variations in the local magnetic field, that in turn alters the local signal. fMRI acquisitions are specifically designed to accentuate this effect in order to make is observable. Unfortunately, local magnetic field variations unrelated to the BOLD mechanism, in particular strong magnetic susceptibility variations due to air/tissue boundaries such as those in the sinus cavities, cause severe non-linear image distortions that effectively alter the location and shape of the affected image volume elements (voxels). This makes even the definition of ’resolution’ problematic, as it is essentially a spatially non-linearly varying function. Such effects are absent from EEG, which is simply a set of receiving electrodes (albeit not without its own source of artifacts) [3]. The SPECTRE reconstruction uses high resolution MRI data acquired with techniques specifically designed to be insensitive to these magnetic susceptibility distortions and thus of very high spatial fidelity.

### Advantages of SPECTRE and future work

The SPECTRE reconstruction of EEG data provides obvious significant advantages over fMRI in temporal resolution, since EEG data has very high intrinsic temporal resolution (~ 1*ms*) necessary to capture rapidly varying electric field variations. Moreover, the SPECTRE algorithm can specify what frequency ranges to interrogate, providing a highly flexible analysis framework for focussed investigation of particular frequency bands of interest. On the contrary, even rapid fMRI acquisition is intrinsically limited by the temporal evolution of the contrast mechanism, the BOLD signal, which is related to blood flow and thus of quite low frequency (~ 1*Hz*).

In this paper, we have successfully validated the SPECTRE method using simultaneous fMRI/EEG experiments. The results not only affirmed SPECTRE’s capability to accurately reconstruct spatial distributions of neural activity from EEG data, in alignment with the concurrently acquired fMRI data, but also revealed its efficacy in identifying robust activations across subjects that were not detectable with fMRI alone. These findings underscore SPECTRE’s potential to significantly enhance the sensitivity and scope of neuroimaging analyses. Further validation was performed using intra-cranial EEG measurements from an epilepsy study with reconstruction of data from a subset of sensors on the surface of the brain were shown to be consistent with the reconstruction from all the sensors, including those directly next to the activity source. The application of SPECTRE to high resolution EEG data during a gambling task demonstrated its ability to reconstruct a well-known and important brain circuit [48–50, 52, 59, 60, 80] that has previously only been detected using fMRI. The analysis revealed significant differences in the brain networks in the alpha range 8 − 12*Hz*, consistent with previous spatially resolved fMRI experiments but the analysis is easily carried out in any user-defined frequency ranges of interest [81–84], which will be the subject of future work. The SPECTRE methodology is applicable to any EEG study and thus holds promise for a wide range of ongoing studies of basic neuroscience of reward mechanisms and in clinical applications such as addiction.

### Conclusion

The implications for spatially resolved EEG are important not only from a scientific perspective, but from a practical perspective as well. fMRI is a much more involved and expensive procedure, requiring highly trained research or clinical applications specialists in specially designed facilities, and subjecting the subjects to a much more claustrophobic and restricted environment, with the safety concerns always present in MRI imaging experiments. On the contrary, the portability, safety, and relative ease of EEG experiments, which can be carried out in a standard research or clinical office, makes it very attractive. The high spatial and temporal resolution capabilities provided by SPECTRE to standard EEG data offer the possibility of more detailed investigations of brain activity in a wide range of both basic research and clinical settings. This method also has important implications for the democratization of medicine worldwide where there are many populations for which advanced technologies such as fMRI are prohibitive because of cost, citing issues for large specialized equipment, and lack of highly trained personnel.

## Figures and Tables

**Figure A.14:**
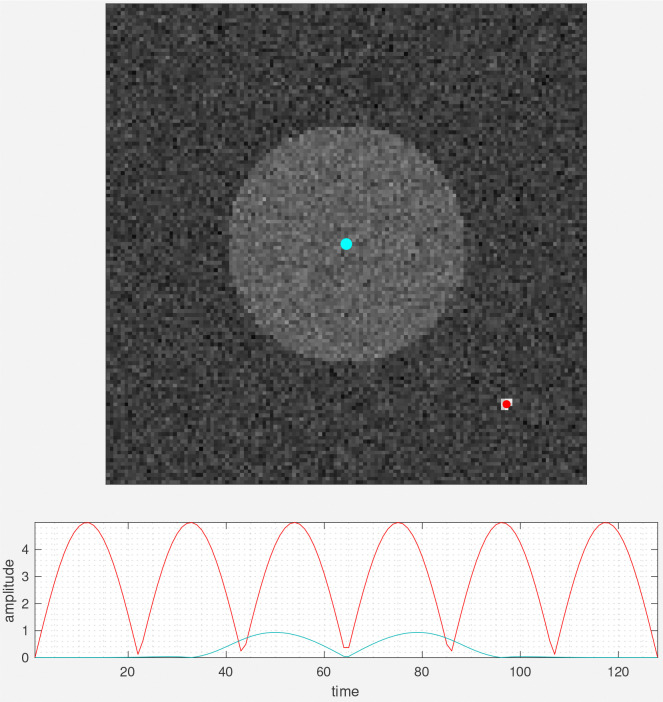
The significance of a detected spatiotemporal pattern. Idealized numerical simulation in which there are two areas of activity in Gaussian random noise: a point oscillating with very high signal-to-noise (SNR) (centered on the red dot) but also a larger circular region with very low SNR (centered at the blue dot). Traditional estimation methods tend to favor high SNR signals (red dot) but have difficulty with low SNR activity with very high spatial correlations (blue dot). The EFD takes both spatial and temporal correlations into account and therefore detects both regions.

**Figure A.15:**
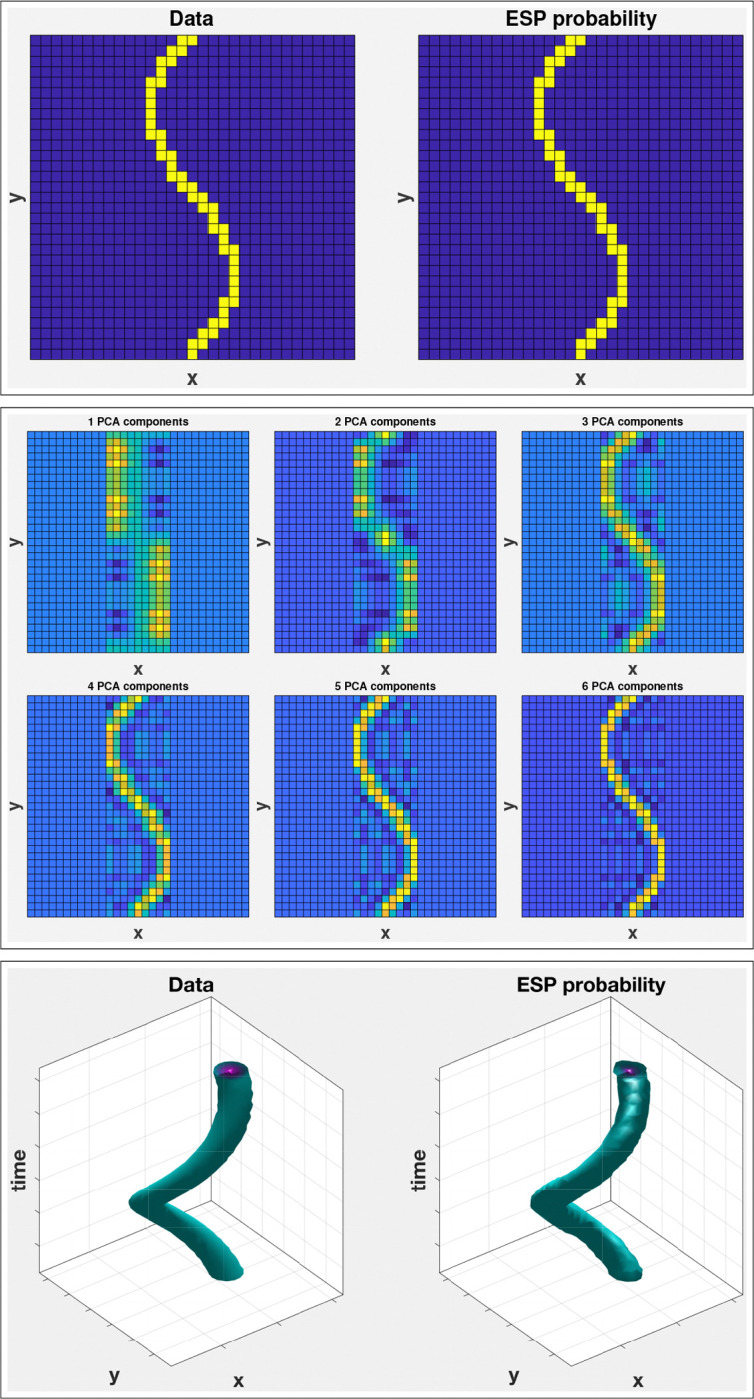
(Top left) Original lattice; (Top right) ESP probability. (Middle) Principal Components Analysis (PCA) results on same original lattice. The ESP probability locates the structure in a single calculation. The PCA decomposition even for 6 components shows significant errors. Several more components would be required to accurately fit the data. (Bottom row) Space-time ESP. (Top left) Space-time trajectory of 2D data; (Top right) ESP probability.

**Figure A.16:**
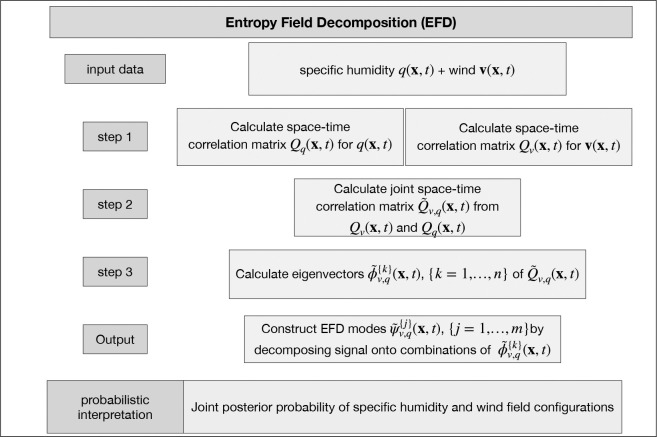
Schematic for the construction of space-time EFD modes from the data.

**Figure B.18:**
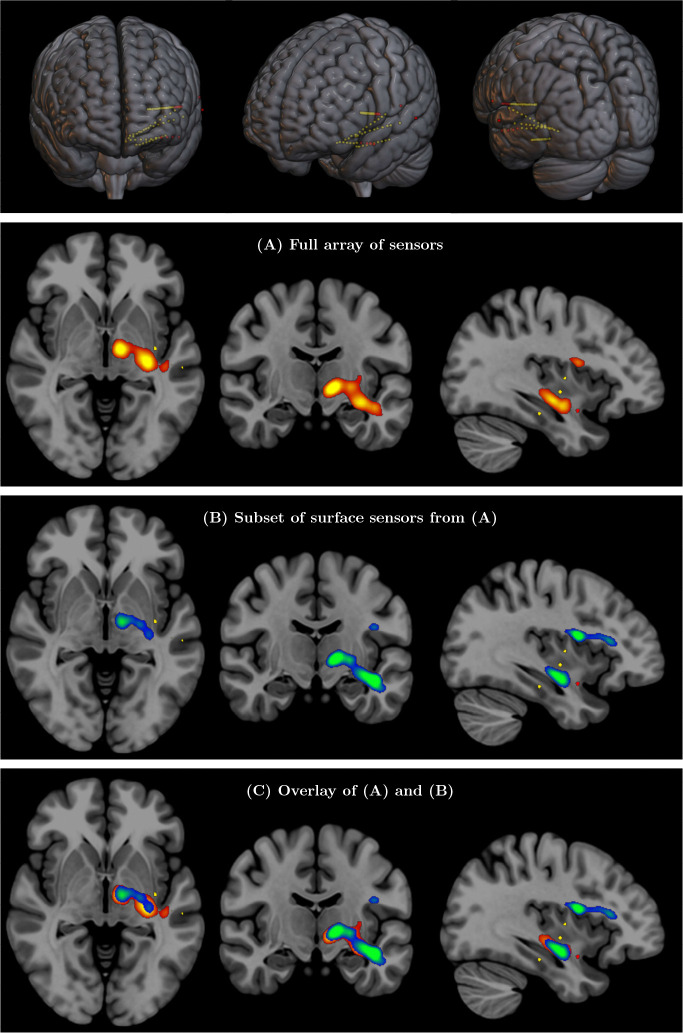
Validation of SPECTRE with iEEG data for subject 3 (see Fig.8 for details.)

**Figure B.19:**
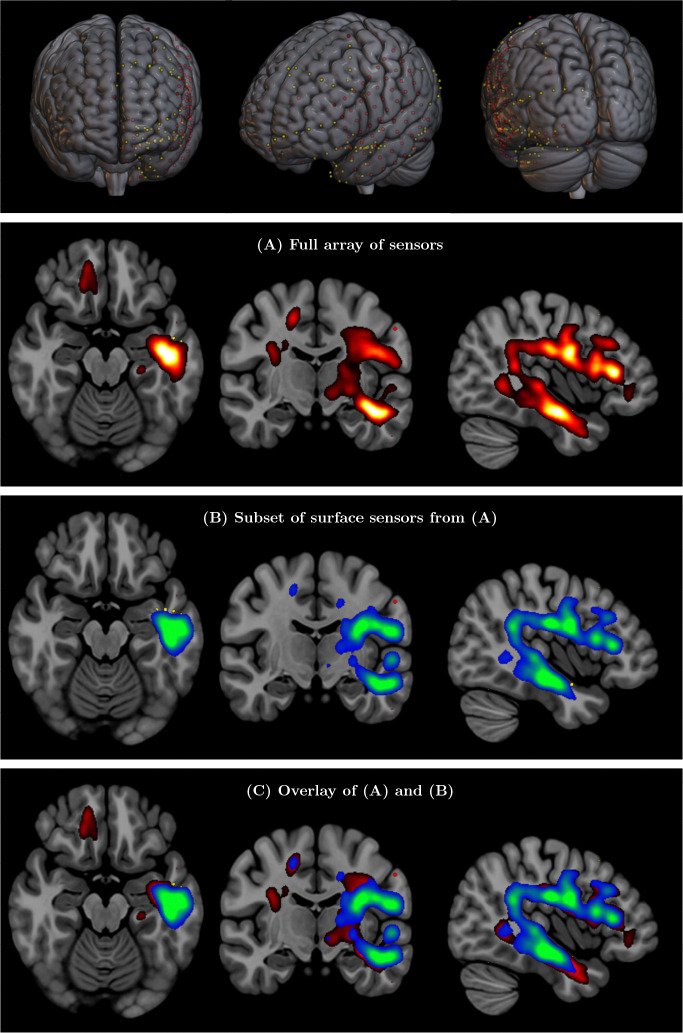
Validation of SPECTRE with iEEG data for subject 4 (see Fig.8 for details.)

## Data Availability

The data that support the findings of this study are available from the corresponding author upon reasonable request.

## Appendix A. Appendix: Entropy Field Decomposition

### Appendix A.1. Inverse problems in dynamical systems

Analysis of weather systems can be considered from two viewpoints. One view is the theoretical construction of the equations governing atmospheric dynamics that when implemented computationally facilitates numerical exploration of the effects of different meteorological parameters on the final atmospheric configurations. This is the *forward problem*. The other view is attempting to reconstruct the actual weather system that was observed with real measured data. This is the *inverse problem*. These viewpoints are complementary, as the comparison of measurements with predicted results from theoretical models is the basis for the refinement of theoretical models of the atmosphere, while the incorporation of more refined models into the reconstruction problem can produce more accurate quantitative assessments of actual events.

However, exceedingly complex non-linear multivariate physical systems pose unique challenges, and the Earth’s weather is a prime example. It is a stratified multi-constituent, multi-phase fluid in an external (gravitational) field with an irregular, multi-phase, rotating lower boundary (the Earth’s surface). Consequently, atmospheric phenomena happen on a broad range of spatial and temporal scales, from planetary waves to severe local storms, and no theoretical construct can hope to precisely capture all the complexity of atmospheric dynamics. But even if this were possible, there is another significant confound: Non-linear systems are exquisitely sensitive to initial conditions in which small changes can result in vastly different final states. Indeed, it was in the study of weather where such “chaotic” systems were brought to widespread attention [85, 86] This means that even with well-defined governing equations the final state cannot be exactly predicted. Therefore the forward problem of simulating the weather from both idealized equations and unknown initial conditions will always be only an approximation, from an analytical perspective, and unpredictable with respect to initial conditions. Thus it is essential to have a general framework with which to address the inverse problem of characterizing atmospheric dynamics from *actual data* from meteorological measurements, which are the result of all the exact physical processes and initial conditions that actually occurred, however unknown.

However, solving inverse problems from data acquired from measurements of non-linear systems is a well-known challenge in many, if not most, scientific disciplines where real data are collected. Neuroimaging data is particularly challenging because it varies over so many spatial and temporal scales and has so many interacting parameters (e.g., electric and magnetic fields, local metabolism, blood flow at arterial, vascular, and capillary scales, etc). This is in addition to the usual challenges of inverse problems, such as finite sampling and both instrument and environmental noise. Indeed, the analysis of dynamic non-linear systems poses a very general analysis problem across multiple scientific disciplines.

### Appendix A.2. Probabilistic approach

Characterizing the complex dynamics of a physical system by estimating the parameters of a hypothesized physical model falls under the purview of probability theory and was the motivation for our development of the entropy field decomposition (EFD), described in detail in [23, 24]. Here we present a more intuitive overview (based on [23]), and limit the discussion to details pertinent to meteorological data.

The meteorological data we are interested in are 4-dimensional, sampled at 3 spatial dimensions and one time dimension. The spatial sampling is typically $\left\{n_{\mathrm{lat}},n_{\mathrm{lon}},n_{\mathrm{lev}}\right\}$ different latitudes, longitudes, and elevations, respectively. So the total number of points in a volume is $N=n_{\mathrm{lat}}\times n_{\mathrm{lon}}\times n_{\mathrm{lev}}$. Measurements from this volume are made at a set of discrete times $t_{i}$, i=1,…,n (assumed to be at equal intervals, though this is not a requirement of the analysis.) The data $\left\{d_{i,j}\right\}$, i=1,…,n, j=1,…,N can equivalently be represented as $\left\{d_{l}\right\}$, where $\xi_{l},l=1,\ldots nN$ defines a set of space-time locations. Each data point is of the form

(A.1)
$$d_{i,j}=Rs_{i,j}+e_{i,j}$$

where R is an operator that represents the response of the measurement system to a signal $s_{i,j}$, and $e_{i,j}$ is the noise with the covariance matrix $\Sigma_{e}=\left\langle ee^{†}\right\rangle$ where † means the complex conjugate transpose.

In NWP, the model equations are run forward in time using a set of initial conditions to generate the signal s, from which can be constructed data d by finite sampling the generated signal and adding noise and instrument constraints. For the inverse (or estimation) problem, the goal is to instead determine the signal s from the actual measured data d. This procedure is codified using Bayes Theorem to construct the posterior probability of the signal s, given the data d, and any information I that is known about the problem, including any assumptions, or hypotheses H:

(A.2)
$$\underset{\mathrm{Posterior}}{\underbrace{p(s|d,I,H)}}=\frac{\overset{\mathrm{Likelihood}}{\overbrace{p(d|s,I,H)}}\;\overset{\mathrm{Prior}}{\overbrace{p(s|I,H)}}}{\underset{\mathrm{Evidence}}{\underbrace{p(d|I,H)}}}$$

The most probable signal is the one reconstructed from the configuration of estimated parameters to produce the maximum in this posterior distribution. The underlying signal is assumed to be continuous in space-time, and thus characterized by a field ψ(x,t)≡ψ(ξ) although the data consist of discrete samples in both space and time so that the reconstructed underlying signal is $s_{l}=\int \psi (\xi )\delta \left(\xi -\xi_{l}\right)d\xi$.

While the Bayesian approach to signal analysis is not new (For an excellent introduction, see [87]), there have remained practical issues that have limited its applicability to non-linear interacting fields. By interacting we are referring to fields that influence one another. For instance, in fMRI blood flow at multiple scales (e.g., vascular and capillary) and electrophysiology are coupled and influence each other. The limitations in practical applications can be thought of as falling into two broad categories: 1) How to represent non-linear interacting fields and 2) How to incorporate relevant prior information. One approach to the first problem is reformulating Bayes Theorem in terms of field theory [88], called *information field theory* (IFT), which facilitates the use of techniques developed in the physics discipline of field theory (e.g., [89]). The advantage, and disadvantage, of this method is that it is essentially flexible in its ability to approximate any desired number of parameters and field interactions. This reformulation can be done by rewriting Bayes Theorem ((A.2)) in the form [88]

(A.3)
$$p(\psi |d,I)=\frac{e^{-H(d,\psi )}}{Z(d)}$$

where the field theoretic quantities, the Hamiltonian H(d,ψ) and the partition function Z(d), are

(A.4a)
H(d,ψ)=-lnp(d,ψ∣I)

(A.4b)
$$Z(d)=p(d|I)=\int d\psi \;e^{-H(d,\psi )}$$

The partition function is a *generating function* from which can be constructed expressions for all orders of field interactions. For the current purposes, however, it will be considered a constant that can be ignored. The Hamiltonian describes the conserved quantities in field theories, and in IFT describes the conservation of probability. This will be the central quantity of interest. It is written

(A.5)
$$H(d,\psi )=H_{0}-j^{†}\psi +\frac{1}{2}\psi^{†}D^{-1}\psi +H_{i}(d,\psi )$$

where $H_{0}$ is essentially a normalizing constant that can be ignored, D is an information propagator, j is an information source, and $H_{i}$ is an interaction term (Eqn 7 in [23]). The solution for the fields is the minimum of the Hamiltonian (δH/δψ=0) is

(A.6)
$$\psi (\xi )=D\left(j-H_{i}^{\prime }\right)$$

where $H_{i}^{\prime }$ symbolically represent the variation if $H_{i}$ (the second term on the RHs of Eqn 8 in [23]).

The expressions (A.5) and (A.6) are not in a form clearly equivalent to the more standard formulations of probability theory, so it is useful to demonstrate their equivalence in a simple problem. This will then make apparent where it deviates from standard formulations for use in more complex problems.

Consider the simple case in which the signal is assumed to be described by a set of model functions F, with each component weighted by an amplitude a. If the functions F do not interact with one another or the noise, the interaction term $H_{i}^{\prime }$ in A.6 can be ignored. Ignoring instrument effect (R=1) and assuming the signal is contaminated by zero mean Gaussian noise with variance $\Sigma_{e}=\sigma^{2}$, the solution is [23]).

(A.7)
$$\psi (\xi )=Dj\;\mathrm{where}\;\left\{\begin{array}{l} D=\sigma^{2}{\left(F^{†}F\right)}^{-1}\\ j=\sigma^{-2}F^{†}d \end{array}\right.$$

The source j is noise weighted projection of the signal onto the sampled model functions (sometimes called the "dirty map" [90]), and the propagator D characterizes the influence of the noise and the sampling of the functions F (sometimes called the "dirty beam" [90]). The estimated signal is

(A.8)
$$\psi (\xi )=F^{+}d\;\mathrm{where}\;F^{+}={\left(F^{†}F\right)}^{-1}F^{†}$$

where $F^{+}$ is the well-known pseudo-inverse. (A.8) is just the standard maximum a posterior result [91]. The rationale for the names "source" and "propagator" becomes clear. The input data $d\left(t_{i}\right)$ projected along the kth component of the model function $F\left(t_{i}\right)$ provides the source j of new information, which is then propagated by D from which estimates of the field components are derived.

If the physical system were describable in terms of non-interacting plane waves, the F would be the standard Fourier functions, then the source is just the noise weighted inverse Fourier transform of the data while the propagator is the covariance of the sampled Fourier model functions. Each Fourier component would constitute a “mode” of the system, which would be a 4-dimensional (3 spatial and 1 temporal) time varying volume. These modes would be ranked according to their amplitude, or eigenvalue. This result is called the “Fourier decomposition” of the data and is ubiquitous across a wide range of scientific disciplines. It is important to note that this Bayesian analysis reveals that the estimate of the spatiotemporal patterns is *not* just the inverse Fourier transform of the data, but takes into account the uncertainties related to the sampling and the noise via the propagator D.

Unfortunately, many, perhaps most, physical systems are not easily characterized by such simple models. This is certainly the case for the planetary weather systems considered in this paper, where a more appropriate general model is that of non-linear, non-periodic, interacting fields. This makes the process of estimating the system from the data much more difficult, because many more combinations of the model parameters are consistent with the data. In addition, non-linear systems can be exquisitely sensitive to initial conditions, which are rarely known precisely and again can produce a wide range of results for the same set of system parameters. The IFT provides a framework to formally characterize complex interacting non-linear fields, but no guidance on resolving these ambiguities. The resulting field theoretic description produces expressions with an essentially infinite number of terms. Without a method for effectively ranking the relative importance of the terms, problems can quickly become intractable.

### Appendix A.3. The role of prior information

Solving seemingly intractable inverse problems requires the reduction in the number of possible system configurations (i.e., the set of parameters characterizing the system) that are consistent with the data. This can be achieved through the development of increasingly refined theoretical physical models. But probability theory provides another powerful mechanism, which is the ability to incorporate prior information. Formally, this is done through the “prior” in (A.2). However, the apparent simplicity of this term belies the complexity in using it. One common and conceptually simple use is to incorporate statistical parameters from previous measurements that characterize a distribution known, or estimated, to describe the system. For example, for a system whose parameters are assumed to be normally distributed, estimates of average and standard deviation can be used to construct a Gaussian prior distribution. While such an approach is common, it is not particular useful when systems are highly non-linear and non-Gaussian.

One of the remarkable traits of the Earth’s atmospheric dynamics is that, despite its seemingly chaotic nature, it displays the remarkable ability to produce phenomena that are highly coherent in space and time, such as planetary waves, hurricanes, and tornadoes. Characterizing such coherences is one of the major objectives of meteorological data analysis. Observations of such data bring up a subtle but important issue that arises in the estimation of coherent space-time processes. By coherence we mean that there are spatial patterns that are persistent in time. For example, a tornado is a persistent spatially localized vorticity. While these notions are intuitively clear, they are notoriously difficult to formalize in analytical theories of estimation. Consider for example the idealized numerical simulation shown in Fig.A.14 in which there are two areas of activity in Gaussian random noise: a point oscillating with very high signal-to-noise (SNR) (centered on the red dot) but also a larger circular region with very low SNR (centered at the blue dot). The computation question can be phrased in terms of “What is the significance of the activated regions?” In more intuitive terms, while the high SNR region clearly seems to indicate an area of activity, we intuitively understand that the larger region is significant despite its low amplitude because it is so persistently coherent over such a large spatial region. Is there a way to incorporate the spatial coherence into the computation of significance?

### Appendix A.4. Entropy Spectrum Pathways (ESP)

The problem then is this: How does one identify regions of spatial and temporal coherence in a dataset that contains complex spatiotemporal patterns that are unique from event to event and thus not amenable to fitting by some standard model? This was the problem faced by Lorenz who recognized that the implications of the unpredictability of atmospheric systems for estimation theory required a general method that could be applied to any dataset from such systems, which led him to the formulation of *empirical orthogonal functions* (**EOF**) [92], which is synonymous with the *principal components analysis* (**PCA**) [93]. On the face of it, PCA is “model free” in that the physical model is not parameterized. However, it is based on the assumption that the data can be described by a multivariate Gaussian distribution and proceeds by successively fitting the data to n ellipsoids following the subtraction of the previous n-1 components. While PCA is easy to implement, it typically is a poor model for complex physical systems and the resulting components therefore can often have little relationship to the actual coherent modes within the data, and therefore makes identification of physically relevant parameters more problematic.

Remarkably, one answer to this question of how to incorporate prior information on system coherences is hidden in plain sight - it is contained within the data itself. Spatial and temporal coherences are characterized by being significantly correlated with their neighbors. In an image, this could simply be the similarity of intensities in neighboring spatial locations. In meteorological data, it might take the form of similar velocity vectors in neighboring regions of both space and time. This concept can be formalized by computing space-time correlations from the data, which are local interactions (i.e., computed in adjacent spatial and temporal points in the data) but, when computed at every location in a dataset, also provide information about the larger scale coherent structures in the data. The relationship between these local interaction and the large scale structures in the data is subject of the theory of *entropy spectrum pathways* (**ESP**) [38].

The essence of the theory is that the coupling between adjacent (in space or time) data elements i and j can be viewed as a measure of the information that can be transmitted between those two locations. One could imagine computing the correlation between the copper content in adjacent elements of a volumetric image of a transmission cable. The resulting high probability regions of adjacent high correlations would be the copper wire, and therefore literally be the path of maximum information transmission. This concept can be formalized and abstracted to any dataset using and parameter or set of parameters. The local interactions reveal the large scale pathways in the data and therefore the pathways of information transmission about the coherences of these parameters. This concept is formalized by computing a *coupling matrix* $Q_{ij}$ between adjacent data elements i and j and computing its eigenvectors $\phi^{\left(k\right)}$, k=1,…,M. (For a detailed discussion of the construction of $Q_{ij}$, see [23].) Remarkably, these eigenvectors can be interpreted as the pathways between locations that can be reached in the most number of ways. In other words, the path in the data that have maximum path entropy. In the wire example, an electron starting at any location on the wire would preferentially travel along that wire. The eigenvector with the largest eigenvalue (k=1), the principal eigenvector, defines the maximum entropy pathway, that which is most preferred.

A simple example of ESP to uncover spatial correlations is shown in Fig.A.15 (Top) where a simple 2D spatial curve is detected with the principal eigenvector of the coupling matrix formed from the intensities at each location. This is to be compared with the standard PCA result in Fig.A.15 (Middle) that required multiple components to be computed and still only approximately detects the correct structure. The extension to space-time is shown in Fig.A.15 (Bottom) where the principal ESP eigenvector correctly picks out the preferred space-time pathway. PCA in this case (not shown) becomes even more problematic.

The recognition that non-Gaussian systems are poorly described by PCA leads to the formulation of a non-linear version called *independent components analysis* (**ICA**) which seeks to decompose data into independent non-Gaussian components. However, this “model free” method has also contains hidden assumptions that produce unreliable results in non-linear complex physical systems (see [24] for a more extended discussion and examples).

### Appendix A.5. The entropy field decomposition

The ESP prior can be incorporated into the estimation scheme by using the coupling matrix $Q_{ij}$ as the prior in (A.2)

(A.9)
$$p(s|d,I)=\frac{1}{|2\pi Q{|}^{1/2}}exp\left(-\frac{1}{2}s_{i}^{†}Q_{ij}s_{j}\right)$$

which then becomes part of the Hamiltonian in (A.3). (For a detailed discussion of the construction of $Q_{ij}$, see [23].) The result is that the significant spatial and temporal correlations within the data can provide highly relevant prior information about the most probable parameters configurations of a physical system, and select out only the limited number of relevant terms within the infinite number of choices that make the inverse problem consistent with the data. The incorporation of the ESP theory into the Bayesian framework of IFT is called the *entropy field decomposition* (**EFD**).

Returning to the simple example above, with the ESP prior, the solution given in (A.7) is the same but with a modified propagator that now includes the coupling matrix:

(A.10)
$$D={\left[Q+F^{†}\Sigma_{e}^{-1}F\right]}^{-1}$$

This means that the estimated modes of the system are now dependent on the coupling matrix Q. And in particular, the optimal solutions depend on the eigenvectors ϕ of Q. Therefore the EFD modes are found by expressing the probability, that is, the Hamiltonian, in terms of ϕ. In other words, whereas in the simplest, uncoupled problem above the Fourier functions serve as the proper ’basis’ to describe the problem, now the eigenfunction of the Q serves that function. These eigenvectors incorporate both the spatial and the temporal correlations, and thus address the problem shown in Fig.A.14: the significance of the detected activated region is enhanced by the adjacency, or clustering, of the activated voxels, as we intuitively believe. Therefore, no post-hoc clustering of voxels determined independently to be activated based on their time course is required, which is the current widespread methodology for detecting activation in, for example, functional MRI (fMRI) data [94–97].

This extends to the more general solution where interactions between fields are considered as well and the interaction Hamiltonian $H_{i}^{\prime }$ (A.6)) is not ignored (see [23] for details). In general, solving the eigenvalue problem for Q produces an *transition probability* $p_{ijk}$ between locations (i,j) for the k’th mode and an *equilibrium distribution* $\mu^{\left(k\right)}$ associated with the set of k=1,M mode eigenvectors $\phi^{\left(k\right)}$. The EFD procedure produces M modes that are ranked by their eigenvalues, providing a simple, unsupervised method for characterizing the primary modes of data collected from an arbitrarily complex non-linear, non-periodic, non-Gaussian physical system.

A compelling feature of the EFD approach is that the basis functions *are unique to every problem*, since they are derived directly from the data. This makes the EFD approach powerful for physical systems that are always unique, such as weather systems. Moreover, the key practical issue is that the coupling matrix Q can be defined by the user according to the problem at hand. The details of incorporating multiple parameters into the coupling matrix are discussed in [35]. A schematic of the EFD procedure is shown in Fig.A.16.

### Appendix A.6. Multi-modality EFD (JESTER)

Extending the EFD methods to multiple modalities by incorporating coupling between different parameters, which we call *Joint Estimation with Entropy Regularization* (JESTER) [35], is accomplished as follows. For m=1,…,M different modalities $d^{\left(m\right)}$ with the coupling matrices $Q^{\left(m\right)}$ that all correspond to the same unknown signal s, intermodality coupling matrix can be constructed as the product of the coupling matrices for the individual modalities expressed in the ESP basis and registered to a common reference frame, which we denote ${\tilde{Q}}^{\left(m\right)}$: That is, the joint coupling matrix is ${𝒬}^{\left(m\right)}=\prod_{m}{\tilde{Q}}^{\left(m\right)}$. More specifically, the joint coupling matrix ${𝒬}_{ij}$ between any two space-time locations (i,j) can be written in the general (equivalent) form as

(A.11)
$$ln\;{𝒬}_{ij}=\sum_{m=1}^{M}\beta_{ij}^{\left(m\right)}ln\;{\tilde{Q}}_{ij}^{\left(m\right)}$$

where the exponents $\beta^{\left(m\right)}$ can either be some constants or functions of data collected for different modalities $\beta_{ij}^{\left(m\right)}\equiv \beta^{\left(m\right)}\left({\tilde{d}}_{i},{\tilde{d}}_{j}\right)$, ${\tilde{d}}_{i}\equiv \{{\tilde{d}}_{i}^{\left(1\right)},\ldots ,{\tilde{d}}_{i}^{\left(M\right)}\}$ where ${\tilde{d}}_{i}^{\left(m\right)}$ and ${\tilde{Q}}_{ij}^{\left(m\right)}$ represent, respectively, the data and the coupling matrix of the modality dataset m represented in the ESP basis and evaluated at locations $r_{i}$ and $r_{j}$ of a common reference domain R:

(A.12)
$${\tilde{d}}_{i}^{\left(m\right)}=d^{\left(m\right)}(\psi^{\left(m\right)}\left(r_{i}\right)),\;{\tilde{Q}}_{ij}^{\left(m\right)}=Q^{\left(m\right)}(\psi^{\left(m\right)}\left(r_{i}\right),\psi^{\left(m\right)}\left(r_{j}\right))$$

where $\psi^{\left(m\right)}\;:R\to X$ denotes a diffeomorphic mapping of m-th modality from the reference domain R to an acquisition space X. In the general EEG reconstruction problem, the coupling of modalities can include the EEG data, the high resolution anatomical MRI data, fMRI data, and diffusion MRI data [21].

## Appendix B. Supplementary Material: Data and Methods

### Data

#### Visual paradigm data

For complete details of the fMRI and EEG acquisitions, see [40]. Minimal details for the EEG and MRI acquisitions here for continuity

##### *EEG acquisitions.* (duplicated from [40]).

EEG is collected using a customized cap to record 61 cortical channels, two electrooculogram (EOG) channels placed above (channel 64) and below the left eye (channel 63), and one electrocardiography (ECG) channel (channel 32) placed on the back. In addition, the cap also contains a reference and ground electrode. Electrodes were filled using V19 Abralyt HiCl electrode gel. Electrode impedance was was kept below 20kOhm. EEG was recorded using BrainVision Recorder at a sampling rate of 5 kHz.

##### *MRI acquisitions.* (duplicated from [40]).

MRI data were acquired using a 12-channel head coil on 3.0 T Siemens TIM Trio. MPRAGE structural T1w images were acquired with the following parameters: TR = 2500 ms; TI = 1200 ms; TE = 2.5 ms; slices = 192; matrix size = 256 × 256; voxel size = 1 mm3 isotropic; flip angle = 8 deg; partial Fourier off; pixel bandwidth = 190 Hz/Px. All BOLD fMRI sequences were acquired with these parameters: TR = 2100 ms; TE = 24.6 ms; Flip Angle = 60°; slices = 38; matrix size = 64 × 64; voxel size = 3.469 × 3.469 × 3.330 mm.

#### Attention paradigm data

##### Simultaneous EEG/fMRI acquisition:.

Functional and structural MRI images were acquired on a Siemens 3T TIM-Trio scanner (NKI Center for Biomedical Imaging and Neuromodulation) equipped with a 32-channel phased array head coil. Structural T1 and T2 scans were collected using standard sequences. Whole-brain BOLD data was acquired with a gradient-echo EPI sequence (TR=2000ms;TE=30ms;flip angle=80°). EEG data were acquired concurrently with fMRI using an MR compatible EEG amplifier (BrainVision MR series, Brain Products, Munich, Germany) and a 64-channel MR-compatible ring electrode cap with 10–20 International System electrode placement cap. EEG data was sampled at a rate of 5 KHz EEG data were acquired at a rate of 5 KHz using BrainVision Recorder software (Brain Products). Electrocardiographic data were captured from electrodes on the backs of subjects. The reference electrode was positioned between Fz and Cz. Scanner and heartbeat artifacts were removed offline from the EEG signal using an average template subtraction procedure [98] and the data was resampled to 250Hz.

##### Traditional EEG analysis:.

The single-trial EEG signal from each electrode was convolved with a 3-cycle Morlet wavelet computed over a 3 second window centered at the onset of each stimulus and averaged separately for each stimulus type. The averaged spectral amplitude at each time point was then baseline corrected by subtracting the mean spectral amplitude over the −200 to −50 pre-stimulus interval. Further details of post-processing and time-frequency analyses methods are described in [99–101].

#### Reward circuit data

##### Task and Design Acquisition.

Participants completed a simple gambling task [64]. On each trial, they saw a black fixation cross for 500 ms, followed by two colored squares for 500 ms, and then, the fixation cross turned gray (go cue) and participants were to select one of the two squares (square locations—left, right—were randomized on each trial) within a 2,000 ms time limit. They were then presented with a black fixation cross for 300 to 500 ms, and then, simple feedback as to their performance (“WIN” for gain, “LOSE” for loss) for 1,000 ms in black font. If the participants responded before the go cue they were instead delivered “TOO FAST” feedback and if they did not respond before the 2,000 ms time limit, it would be considered a loss. The goal of the participants was to accumulate wins by determining which of the two squares would more often lead to gains (60% vs. 10%). In this task, participants accumulated wins; however, were not paid money. They would see the same pair of colors for one block of 20 trials. They conducted six blocks of unique color pairs.

##### Participants.

Five hundred undergraduate students were included and were recruited via the University of Victoria psychology participant pool (see [64] for details). The data was collected until 500 participants became available that were not characterized by one of the following a priori criteria: trial count after artifact rejection were less than 15 per condition, total artifact rejection exceeded 40% of trials rejected, FCz (electrode of interest) specific artifact rejection exceeded 40% of trials rejected, or independent component analysis based blink correction failed. These criteria were extremely strict to ensure clean data in the analyses, and as such a total of 637 participants were analyzed before reaching the goal of 500 clean participants. All participants had normal or corrected-to-normal vision and volunteered to take part in the experiment for extra course credit in a psychology course. All participants provided informed consent approved by the University of Victoria’s Human Research Ethics Board.

##### Data Acquisition and Preprocessing.

EEG data were recorded from either a 64 or 32 electrode (Ag/AgCl) EEG system (ActiCAP, Brain Products, GmbH, Munich, Germany) using Brain Vision Recorder (Version 1.21.0004, Brain Products GmbH, Munich, Germany). During recording, electrodes were referenced to a common ground, impedances were kept below 20 kΩ on average, data were sampled at 500 Hz, and an antialiasing low-pass filter of 245 Hz was applied via an ActiCHamp amplifier (Revision 2, Brain Products GmbH, Munich, Germany). Stimuli and EEG markers were temporally synced using a DataPixx stimulus synchronization unit (VPixx, Vision Science Solutions, Quebec, Canada)

Data were re-referenced to an average mastoid reference and filtered using a 0.1 to 30 Hz passband (Butterworth, order 4) and a 60 Hz notch filter. Correction for eye blinks was performed using EEGLAB’s independent component analysis (ICA). Components reflective of blinks were manually identified and removed via topographic maps and component loadings, and data were reconstructed. Data were then segmented from −500 to 1, 500*ms* relative to feedback stimulus onset, baseline corrected using a −200 to 0*ms* window, and run through artifact rejection with 10*μV/ms* gradient and 100*μV* maximum–minimum criteria. Data were pre-processed to identify noisy or damaged electrodes using artifact rejection trial removal rates for each electrode.

The 1 second of recorded sequence for each "WIN" or "LOSE" event were extracted from recordings for each participants (with 22 ms of pre-event sample and 488 ms of post-event sample) and combined together to form separate winning and loosing datasets. Each of those datasets were processed using SPECTRE to construct the approximate inverse solution for the potential ϕ across an entire 2 mm MNI brain volume.

#### Intra-cranial EEG validation: Additional subjects

The analysis presented in Figs.B.17 to B.19 are the results of same analysis for Subjects 2–4 as presented for subject 1 in Fig.8.
