# Supplementary material for: Imaging of brain electric field networks
Source: Res Sq. 2024 Apr 12:rs.3.rs-2432269. Preprint. [Version 2] doi: 10.21203/rs.3.rs-2432269/v2 (PMC11042417; doi:10.21203/rs.3.rs-2432269/v2)
Supplement: Supplementary file 1 [file NIHPPrs2432269V2-supplement-1.pdf]

6 Supplementary Material: Data and Methods

6.1 SPECTRE details

6.1.1 Solution to the inverse EEG problem

Our method for solving the inverse EEG problem can be summarized as follows. Given a standard EEG dataset from N electrodes and a high-resolution anatomical (HRA) MRI dataset with high contrast between gray matter (GM) and white matter (WM), the solution to the inverse EEG problem can be formulated as an approximation for the volumetric distribution of electrostatic potential inside the complex inhomogeneous and anisotropic tissues and complicated morphology of the MRI domain (*43*). A general form of this approximation is based on Maxwell's equations in an anisotropic and inhomogeneous medium expressed as charge continuity. This is used to derive the frequency dependent electrostatic field potential ϕ that is dependent upon the electrical properties of the tissue permittivity, permeability, and conductivity. These parameters can be estimated from the HRA data. In the Fourier (i.e., frequency) domain, the electrostatic potential satisfies the equation

$$\left(\Sigma^{ij} - I\omega\varepsilon\delta^{ij}\right)\partial_i\partial_j\phi_\omega = \left[I\omega(\partial_i\varepsilon)\delta^{ij} - (\partial_i\Sigma^{ij})\right]\partial_j\phi_\omega + \mathscr{F}_\omega, \tag{1}$$

which is written in tensor form where a summation is assumed over repeated indices. This can be expressed in the form $\hat{L}\phi_\omega = \hat{R}\phi_\omega + \hat{\mathscr{F}}_\omega$ in terms of the operators $\hat{L} \equiv \partial^i\partial_i$, a frequency dependent source term $\hat{\mathscr{F}}_\omega$ and the operator

$$\hat{R} \equiv \frac{\sigma + I\omega\varepsilon}{\sigma^2 + \omega^2\varepsilon^2}\left[I\omega(\partial_i\varepsilon)\delta^{ij} - (\partial_i\Sigma^{ij}) - \left(\Sigma^{ij} - \sigma\delta^{ij}\right)\partial_i\right]\partial_j$$

where $\mathbf{\Sigma} = \{\Sigma^{ij}\}$ is a local tissue conductivity tensor, $\sigma = Tr\mathbf{\Sigma}/3 = \Sigma^i_i/3$ is an isotropic local conductivity. Terms in square brackets show that the parts of $\hat{R}\phi_\omega$ can be interpreted in terms of different tissue characteristics and may be important for understanding the origin of sources of the electro-/magnetostatic signal detected by the EEG sensors. The first term

$(\omega(\partial^i\varepsilon)(\partial_i\phi_\omega))$ corresponds to areas with sudden change in permittivity, e.g. the WM/GM interface. The second term $((\partial_i\Sigma^{ij})(\partial_j\phi_\omega))$ corresponds to regions where the conductivity gradient is the strongest, i.e. the GM/CSF (cerebral spinal fluid) boundary. Finally, the last term $(\Sigma^{ij}\partial_i\partial_j\phi_\omega - \sigma\partial^i\partial_i\phi_\omega)$ includes areas with the strongest conductivity anisotropies, e.g. input from major WM tracts. The frequency and position dependent internal sources $\hat{\mathscr{F}}_\omega$ can be used to incorporate various nonlinear processes including multiple frequency effects of the efficient synchronization/desynchronization by brain waves or effects of their critical dynamics. This term is ignored in the current paper.

The inverse problem can be solved by constructing an approximate solution for the potential ϕ across an entire brain volume iteratively as $\hat{L}\phi_\omega^{(k)} = \hat{R}\phi_\omega^{(k-1)}$ and $\tilde{\phi}_\omega^{(K)} = \alpha_K \sum_{k=0}^{K} \phi_\omega^{(k)}$ (*43*): where a single iteration forward solution is found using a Fourier-space pseudo-spectral approach (*44*). Data from MRI can be used to define the complex brain tissue morphology and constrain the tissue specific values of $\mathbf{\Sigma}$ and ε. This procedure of inverting the WETCOW brain wave model constrained by MRI-defined tissue properties is called *SPatially resolved EEG Constrained with Tissue properties by Regularized Entropy (SPECTRE).*

SPECTRE is flexible in its ability to incorporate relevant prior information from MRI data. HRA data is useful for tissue segmentation and assignment of mean values for permittivity and conductivity. Diffusion MRI (dMRI) data further allows construction of estimates of the conductivity tensor anisotropy. In the present study, only HRA data were used for the estimation procedure. One important practical point is that very often HRA data are not acquired for participants in EEG studies. In this case it is sufficient to use HRA data from a standard atlas, and spatially register the EEG data to the atlas. Indeed, this was case in the present paper, where the standard 2mm, 1mm, and 0.7mm resolution T1-weighted anatomical MRI Montreal Neurological Institute (MNI) (*45*) atlases were used, to which the EEG data were aligned using our non-linear registration algorithm SYMREG (*46*).

6.1.2 Mode reconstruction

The reconstructed volumetric time series of the estimated electrostatic potential field can be thought of as similar to the EM equivalent of an fMRI dataset, containing a multitude of correlated spatiotemporal patterns or "modes" of the system.

The problem then becomes one of detecting the multiple modes in complex non-linear systems. We have addressed this problem previously in our development of the *entropy field decomposition* (EFD) method, which is a probabilistic framework for estimating spatial-temporal modes of complex non-linear systems containing multivariate interacting fields. It is formally based on a field-theoretic mathematical formulation of Bayes' Theorem that enables the hierarchy of multiple orders of field interactions including coupling between fields (*12, 37*). Its practical utility is enabled by incorporation of the theory of entropy spectrum pathways (ESP) (*47*), which uses the space-time correlations in each individual dataset to automatically select the very limited number of highly relevant field interactions. In short, it selects the configurations with maximum path entropy, summarized in the equilibrium (i.e., long time) distribution μ^*.

Our terminology is as follows (see Section 6.1.3 details). The k'th EFD mode of the coupled parameters $\boldsymbol{\alpha} = \{\alpha_1, \ldots, \alpha_m\}$ is denoted $\psi_{\boldsymbol{\alpha}}^{(k)}(\boldsymbol{x}, t)$ where $k = 1, \ldots, n$ for some user defined n. While each of these modes provides unique information on coherent spatio-temporal activity, for characterizing the total brain activity it is often most useful and efficient to sum these modes: $\tilde{\psi}_n = \sum_k^n \psi_{\boldsymbol{\alpha}}^{(k)}(\boldsymbol{x}, t)$. In this study we utilize both individual modes and the summed modes, as appropriate.

A strength of the EFD method is that it uses prior information contained in individual datasets - there are no training datasets or averages across datasets - just the prior information contained within the single dataset of interest. This method has shown utility in resting state fMRI data (*12*) and in meteorology in the application to severe local storms, in particular tornadic supercells (*48*). The fact that this method uses prior information embedded within

single datasets without the need for any ’training’ is of significance to clinical studies in which important individual variations can be lost in the averaging process. It is also particularly important in the current paper where our validation necessitates comparison with single subject studies.

6.1.3 Entropy Field Decomposition

The *entropy field decomposition* (EFD) is a general probabilistic method for the estimation of spatial-temporal modes of complex non-linear, non-period, non-Gaussian multivariate data (*12, 37*). The goal of EFD is to estimate the field $\psi(\boldsymbol{x}, t)$ that describes a continuous (in both space $\boldsymbol{x}$ and time t) parameter space from which the signal s_l are discrete samples of $s_l = \int \psi(\xi)\delta(\xi - \xi_l)d\xi$. In general, this can be done by constructing the posterior distribution of $\psi(\boldsymbol{x}, t)$ given the data d and any prior information I that is available, via Bayes’ Theorem re-expressed in the language of field theory (*49*) as

$$p(\psi|s, I) = \frac{e^{-H(s,\psi)}}{Z(s)} \tag{2}$$

where $Z(d) = p(d|I) = \int d\psi\, e^{-H(s,\psi)}$ is the *partition function* (considered a constant in this application) and $H(s, \psi) = -\ln p(s, \psi|I)$ is the *information Hamiltonian* which takes the form

$$H(s, \psi) = H_0 - j^\dagger\psi + \frac{1}{2}\psi^\dagger D^{-1}\psi + H_i(s, \psi) \tag{3}$$

where H_0 is essentially a normalizing constant that can be ignored, j and D are the information source and propagator, and $\dagger$ means the complex conjugate transpose. H_i is an interaction term (*49*)

$$H_i = \sum_{n=1}^{\infty} \frac{1}{n!} \int \cdots \int \Lambda^{(n)}_{s_1 \cdots s_n} \psi(s_1) \cdots \psi(s_n) ds_1 \cdots ds_n \tag{4}$$

where $\Lambda^{(n)}_{s_1 \cdots s_n}$ terms describe the interaction strength. In highly complex non-linear systems, j, D, and $\Lambda^{(n)}_{s_1 \cdots s_n}$ are often unknown and too complex for deriving effective and accurate approximations. In this case the ESP method (*47*), based on the principal of maximum entropy (*50, 51*),

provides a general and effective way to introduce powerful prior information to find the most significant contributions to $H(d,\psi)$ by using coupling between different spatio-temporal points that is available from the data itself. This is accomplished by constructing a *coupling matrix* that characterizes the relation between locations i and j in the data $Q_{ij} = e^{-\gamma_{ij}}$ where the γ_{ij} are Lagrange multipliers that describe the relations and depend on some function of the space-time locations i and j. The eigenvalues λ_k and eigenvectors $\phi^{(k)}$ of the coupling matrix Q

$$\sum_j Q_{ij}\phi_j^{(k)} = \lambda_k \phi_i^{(k)} \tag{5}$$

then define the transition probability from location j to location i of the k'th mode as

$$p_{ijk} = \frac{Q_{ji}}{\lambda_k}\frac{\phi_i^{(k)}}{\phi_j^{(k)}} \tag{6}$$

For each transition matrix (6) there is a unique stationary distribution associated with each path k:

$$\mu^{(k)} = \left[\phi^{(k)}\right]^2 \qquad \text{where} \qquad \mu_i^{(k)} = \sum_j \mu_j^{(k)} p_{ijk} \tag{7}$$

where $\mu^{(1)}$, associated with the largest eigenvalue λ_1, corresponds to the maximum entropy stationary distribution. Note that (7) is written to emphasize that the squaring operation is performed on a pixel-wise basis.

The essence of the EFD approach (*12,37*) is to incorporate these coupling matrix priors into the information Hamiltonian (3) by expanding the signal $s(\boldsymbol{x},t)$ into a Fourier expansion using $\{\phi^{(k)}\}$ as the basis functions

$$s_i = \sum_k^K \left[a_k\phi_i^{(k)} + a_k^\dagger \phi_i^{\dagger,(k)}\right] \tag{8}$$

which allows expressing the information Hamiltonian (3) in this ESP basis as

$$H(d,a_k) = -j_k^\dagger a_k + \frac{1}{2}a_k^\dagger \Lambda a_k + \sum_{n=1}^{\infty}\frac{1}{n!}\sum_{k_1}^K \cdots \sum_{k_n}^K \tilde{\Lambda}_{k_1\cdots k_n}^{(n)} a_{k_1}\cdots a_{k_n} \tag{9}$$

where matrix Λ is the diagonal matrix Diag$\{\lambda_1, ..., \lambda_K\}$, composed of the eigenvalues of the coupling matrix, and $j_k = \int j\, \phi^{(k)} ds$ is the amplitude of kth mode in the expansion of the source j and the new interaction terms $\tilde{\Lambda}^{(n)}$ are

$$\tilde{\Lambda}^{(n)}_{k_1 \cdots k_n} = \int \cdots \int \Lambda^{(n)}_{s_1 \cdots s_n} \phi^{(k_1)} \cdots \phi^{(k_n)} ds_1 \cdots ds_n \tag{10}$$

from which can be derived (*12, 37*) a simple expression for the solution for the amplitudes a_k

$$\Lambda a_k = \left(j_k - \sum_{n=1}^{\infty} \frac{1}{n!} \sum_{k_1}^{K} \cdots \sum_{k_n}^{K} \tilde{\Lambda}^{(n+1)}_{k k_1 \cdots k_n} a_{k_1} \cdots a_{k_n} \right) \tag{11}$$

through the eigenvalues and eigenvectors of coupling matrix.

The EFD methods can be extended to multiple modalities by incorporating coupling between different parameters, which we call *Joint Estimation with Entropy Regularization* (JESTER) (*52*). For $m = 1, ..., M$ different modalities $d^{(m)}$ with the coupling matrices $Q^{(m)}$ that all correspond to the same unknown signal s, intermodality coupling matrix can be constructed as the product of the coupling matrices for the individual modalities expressed in the ESP basis and registered to a common reference frame, which we denote $\widetilde{Q}^{(m)}$: That is, the joint coupling matrix is $\mathscr{Q}^{(m)} = \prod_m \widetilde{Q}^{(m)}$. More specifically, the joint coupling matrix $\mathscr{Q}_{ij}$ between any two space-time locations (i, j) can be written in the general (equivalent) form as

$$\ln \mathscr{Q}_{ij} = \sum_{m=1}^{M} \beta^{(m)}_{ij} \ln \widetilde{Q}^{(m)}_{ij} \tag{12}$$

where the exponents $\beta^{(m)}$ can either be some constants or functions of data collected for different modalities $\beta^{(m)}_{ij} \equiv \beta^{(m)}(\widetilde{\boldsymbol{d}}_{\boldsymbol{i}}, \widetilde{\boldsymbol{d}}_{\boldsymbol{j}}), \widetilde{\boldsymbol{d}}_{\boldsymbol{i}} \equiv \{\widetilde{d}^{(1)}_i, ..., \widetilde{d}^{(M)}_i\}$ where $\widetilde{d}^{(m)}_i$ and $\widetilde{Q}^{(m)}_{ij}$ represent, respectively, the data and the coupling matrix of the modality dataset m represented in the ESP basis and evaluated at locations r_i and r_j of a common reference domain R:

$$\widetilde{d}^{(m)}_i = d^{(m)}\left(\psi^{(m)}(r_i)\right), \qquad \widetilde{Q}^{(m)}_{ij} = Q^{(m)}\left(\psi^{(m)}(r_i), \psi^{(m)}(r_j)\right) \tag{13}$$

where $\psi^{(m)} : R \to X$ denotes a diffeomorphic mapping of m-th modality from the reference domain R to an acquisition space X.

6.2 Comparison with functional MRI

Functional MRI (fMRI) has become the *de facto* neuroimaging method for spatial and temporal localization of brain activity. The contrast mechanism that forms the basis of fMRI is the blood oxygenation level dependent (BOLD) variations in the magnetic state of hemoglobin and its influence on the local MRI signal as a function of the local metabolism and hemodynamics (*53*). Consequently, the spatial and temporal characteristics of the fMRI signal are related to blood flow and metabolic dynamics, rather than direct measures of electrical activity. In particular, the signal variations will be spatially localized in vascular pathways and the temporal variations, being related to blood flow effects, are very slow compared to electrical activity. In short, the spatial-temporal dynamics measured by fMRI need not (and, in fact, will not) correspond exactly to the spatial-temporal patterns of electrical activity. Numerous experimental realities also make fMRI problematic as a gold standard. In particular, fMRI is facilitated by enhancing the sensitivity of MRI to the BOLD contrast mechanism, which requires enhancing the sensitivity to local magnetic field variations through the use of T_2-weighted pulse sequences (*54*) which lead to increased geometric distortions, compromising not only spatial resolution but confounding the spatial localization of the activity in a complex, non-linear fashion. Gross distortions can lead to significantly reduced signal-to-noise and even completely unrecoverable signal loss, particularly in regions near air/tissue interfaces, such as in the prefrontal cortex (PFC). Moreover, the complex non-linear interactions between the magnetic fields and physiological variations such as respiration and cardiac pulsations produce a variety of complex spatiotemporal signal distortions (*55*). While mitigating these artifacts is an area of very active research, they remain a serious problem for fMRI.

Nevertheless, certain very simple task-based fMRI experimental paradigms, such as finger tapping or rapidly flickering checkerboard stimuli, repeated at periodic on/off intervals, have been established as experiments that produce repeatable robust activations in known brain net-

works, and are commonly used as basic testbeds for assessment of analysis algorithms. When combined with simultaneous EEG acquisition, such experiments provide two different types of data that can be compared as a form of validation, with the proviso that these two methods are imaging different physical quantities.

6.3 Comparison with state-of-the-art Source Localization methods

There is a long history of attempts to spatially localize EEG activity and these are generally called "source localization" methods (*56–59*). These methods are fundamentally different from the SPECTRE approach as they involve numerous stringent assumptions about brain electrical activity such as a fixed set of static dipole sources, an idealized geometric model of the head reduced to a few (typically 3) shells, that spatially close points are more likely synchronized, and the smoothness of the solution. (see (*60*) and references therein).

These methods all implicitly assume the "quasi-static" approximation to the EM field equation which entails ignoring the time dependent terms in Maxwell's equations, which are dependent on tissue conductivity properties which are themselves frequency dependent. The resulting solutions are therefore static, have no frequency dependence, and are insensitive to the detailed spatially variable electrical properties of the tissues. However, as discussed in detail in the development of the WETCOW model (*7–9*), these assumptions are incompatible with the basic physics of brain electrical activity. The SPECTRE approach is to employ the WETCOW model and solve the actual physical problem of the complete Maxwell's equations in an inhomogeneous and anisotropic medium. The WETCOW theory provides a comprehensive framework for characterizing the propagation of EM fields through the complex brain tissue microstructure and larger scale morphology (e.g., cortical folding), and provides the dynamic solution to the electric potential field necessary to solve the EEG inverse problem.

The pseudo-spectral computational approach used in SPECTRE has some important ad-

vantages over the finite/boundary element approaches typically used for electrostatic modeling of brain activity (*61–67*). It does not use surface meshes and so does not require limiting the location of sources to a small number of surfaces with fixed number of static dipole sources constrained to the surfaces. And the distribution of both electrostatic and geometric properties of the media (conductivity, permittivity, anisotropy, inhomogeneity - derived from the MRI data) are incorporated at every location throughout the volume. It is thus able to find a time dependent spatial distribution of the electrostatic potential at every space-time location of a multidimensional volume as a superposition of source inputs from every voxel of the same volume (*43*). These traits allow it to model wave-like signal propagation inside the volume and can detect and characterize significantly more complex dynamical behavior of the sources of the electrostatic activity recorded at the sensor locations than traditional methods.

To understand intuitively why SPECTRE is capable of reconstructing EM activity through the entire brain, including deep within subcortical structures, a simple idealized example is helpful. Consider two point current sources of different frequencies, one in the cortical layer close to the scalp, the second deep within the subcortical structures of the brain. Consider a single sensor placed on the scalp collinear with the two sources. Standard source localization methods will not see the deep source, since there is no frequency dependence, and the signal falloff is simply a function of the distance from the sensor. Therefore, the close source completely dominates the signal model. Since all tomographic imaging methods (e.g., MRI, CT, etc.) depend strongly on both the spatial and temporal sampling of the measured physical system, this effective invisibility of currents in the standard quasi-static model essentially precludes the solution of the true inverse EEG problem and necessitates the artificial construction of assumed dipole distribution on pre-chosen artificial internal structures. In contrast, in SPECTRE the sources are not dipoles, but frequency sources that extend throughout the entire brain volume subject to the boundary conditions imposed by both the tissues geometry and its spatially and frequency

dependent properties. The surface electrodes are assumed to be sensing EM waves emanating from the entire brain across a broad frequency spectrum limited only by the sensors. Used in conjunction with an HRA MRI data that provides the spatial distribution of the frequency-dependent tissue electrical properties that constrain the possible solution, SPECTRE can invert the wave equations to provide an estimate of the spatiotemporal distribution of the electric field potential.

The problem of spatially localizating the EEG signal involves estimating the most probable distribution of electric field amplitudes given an array of sensors. This is essentially a problem of correctly modeling the physics of how electromagnetic waves propagate through the complex environment of a the convoluted brain tissue morphology and the anisotropic and inhomogeneous nature of brain tissue. The current state-of-the-art approach to this problem, called 'source localization' such as *low resolution electromagnetic tomography* or LORETA algorithm with its many variations (*56–59*), also called 'EEG source imaging' (*68, 69*)) involves using a pre-defined brain atlas, arbitrarily placing dipole source on the surface, and calculating the contribution from these sources. Some methods propose using fMRI as a prior, which has the disadvantage of requiring fMRI acquisitions (*70–73*). The current source localization methods are based on a static model for the electric field caused by a fixed set of pre-defined dipole sources. (see (*2*) for a review of current methods). This model is inherently limited because in reality the brain's electrical field variations are time dependent and generated by an essentially continuous distribution of sources throught the entire brain. This description is the essence of the WETCOW theory (*7–9*) which describes how highly coherent localized electric field phenomena, such as cortical wave loops and synchronized spiking, are produced by the complex non-linear interactions of waves across multiple spatial and temporal scales. In a typical application of the SPECTRE method, we use MNI volumetric grid with 2mm (902629 voxels), 1mm (7221032 voxels), or 0.73mm (11393280 voxels). All voxels in our models are

considered sources of electromagnetic activity consistent with the local intravoxel tissue characteristics (via (1)) rather than an assumed dipolar form. In an attempt to compare SPECTRE with current state-of-the-art source localization method, we downloaded the currently available LoretaKey1 software (*59*), which uses a set of 6239 fixed dipoles. In order to make a fair comparison, we tried to use LoretaKey1 with a number of dipoles comparable to our $2mm$ number of voxels. We found that LoretaKey1 is not able to handle this size due to "out-of-memory" crashes. We scaled down the number of dipoles in 2, 4 , 10 and 20 times. Only with around 45K dipoles were we able to make Loreta run. It ran for approximately 24 hours, but then again crashed due to out-of-memory problems. Our processing with 2mm, requires around 650Mb of memory. At this point it was decided that it was not possible for Loreta to provide a result that would usefully inform the efficacy of the SPECTRE method. Our highest 0.73 resolution processing can be completed on a modern workstation taking 16-20Gb of memory in a matter of minutes.

6.4 Data

6.4.1 Attention paradigm data

Simultaneous EEG/fMRI acquisition: Functional and structural MRI images were acquired on a Siemens 3T TIM-Trio scanner (NKI Center for Biomedical Imaging and Neuromodulation) equipped with a 32-channel phased array head coil. Structural T1 and T2 scans were collected using standard sequences. Whole-brain BOLD data was acquired with a gradient-echo EPI sequence (TR=2000ms;TE=30ms;flip angle=80°). EEG data were acquired concurrently with fMRI using an MR compatible EEG amplifier (BrainVision MR series, Brain Products, Munich, Germany) and a 64-channel MR-compatible ring electrode cap with 10–20 International System electrode placement cap. EEG data was sampled at a rate of 5 KHz EEG data were acquired at a rate of 5 KHz using BrainVision Recorder software (Brain Products). Electrocardiographic data

were captured from electrodes on the backs of subjects. The reference electrode was positioned between Fz and Cz. Scanner and heartbeat artifacts were removed offline from the EEG signal using an average template subtraction procedure (*74*) and the data was resampled to 250Hz.

Traditional EEG analysis: The single-trial EEG signal from each electrode was convolved with a 3-cycle Morlet wavelet computed over a 3 second window centered at the onset of each stimulus and averaged separately for each stimulus type. The averaged spectral amplitude at each time point was then baseline corrected by subtracting the mean spectral amplitude over the -200 to -50 pre-stimulus interval. Further details of post-processing and time-frequency analyses methods are described in (*75–77*).

6.4.2 Reward circuit data

Task and Design Acquisition Participants completed a simple gambling task (*36*). On each trial, they saw a black fixation cross for 500 ms, followed by two colored squares for 500 ms, and then, the fixation cross turned gray (go cue) and participants were to select one of the two squares (square locations—left, right—were randomized on each trial) within a 2,000 ms time limit. They were then presented with a black fixation cross for 300 to 500 ms, and then, simple feedback as to their performance ("WIN" for gain, "LOSE" for loss) for 1,000 ms in black font. If the participants responded before the go cue they were instead delivered "TOO FAST" feedback and if they did not respond before the 2,000 ms time limit, it would be considered a loss. The goal of the participants was to accumulate wins by determining which of the two squares would more often lead to gains (60% vs. 10%). In this task, participants accumulated wins; however, were not paid money. They would see the same pair of colors for one block of 20 trials. They conducted six blocks of unique color pairs.

Participants Five hundred undergraduate students were included and were recruited via the University of Victoria psychology participant pool (see (*36*) for details). The data was collected until 500 participants became available that were not characterized by one of the following a priori criteria: trial count after artifact rejection were less than 15 per condition, total artifact rejection exceeded 40% of trials rejected, FCz (electrode of interest) specific artifact rejection exceeded 40% of trials rejected, or independent component analysis based blink correction failed. These criteria were extremely strict to ensure clean data in the analyses, and as such a total of 637 participants were analyzed before reaching the goal of 500 clean participants. All participants had normal or corrected-to-normal vision and volunteered to take part in the experiment for extra course credit in a psychology course. All participants provided informed consent approved by the University of Victoria's Human Research Ethics Board.

Data Acquisition and Preprocessing EEG data were recorded from either a 64 or 32 electrode (Ag/AgCl) EEG system (ActiCAP, Brain Products, GmbH, Munich, Germany) using Brain Vision Recorder (Version 1.21.0004, Brain Products GmbH, Munich, Germany). During recording, electrodes were referenced to a common ground, impedances were kept below 20 kΩ on average, data were sampled at 500 Hz, and an antialiasing low-pass filter of 245 Hz was applied via an ActiCHamp amplifier (Revision 2, Brain Products GmbH, Munich, Germany). Stimuli and EEG markers were temporally synced using a DataPixx stimulus synchronization unit (VPixx, Vision Science Solutions, Quebec, Canada)

Data were re-referenced to an average mastoid reference and filtered using a 0.1 to 30 Hz passband (Butterworth, order 4) and a 60 Hz notch filter. Correction for eye blinks was performed using EEGLAB's independent component analysis (ICA). Components reflective of blinks were manually identified and removed via topographic maps and component loadings, and data were reconstructed. Data were then segmented from -500 to $1,500ms$ relative to

feedback stimulus onset, baseline corrected using a -200 to $0ms$ window, and run through artifact rejection with $10\mu V/ms$ gradient and $100\mu V$ maximum–minimum criteria. Data were pre-processed to identify noisy or damaged electrodes using artifact rejection trial removal rates for each electrode.

The 1 second of recorded sequence for each ”WIN” or ”LOSE” event were extracted from recordings for each participants (with 22 ms of pre-event sample and 488 ms of post-event sample) and combined together to form separate winning and loosing datasets. Each of those datasets were processed using SPECTRE to construct the approximate inverse solution for the potential ϕ across an entire 2 mm MNI brain volume.
